# LOC730101 improves ovarian cancer drug sensitivity by inhibiting autophagy-mediated DNA damage repair via BECN1

**DOI:** 10.1038/s41419-024-07278-1

**Published:** 2024-12-18

**Authors:** Yancheng Zhong, Yang Shuai, Juan Yang, Mojian Zhang, Tiantian He, Leliang Zheng, Sheng Yang, Shuping Peng

**Affiliations:** 1 The Key Laboratory of Carcinogenesis and Cancer Invasion of the Chinese Ministry of Education, Xiangya Hospital and School of Basic Medical Science, Central South University, Changsha, Hunan China; 2 Hunan Key laboratory of Vascular Biology and Translational Medicine, Medical School, Hunan University of Chinese Medicine, Changsha, China; 3 Cancer Research Institute, Central South University, Changsha, Hunan China; 4 College of Life Science and Technology, Huazhong University of Science and Technology, Wuhan, China; 5 Department of Gynecologic Oncology Ward 5, Hunan Cancer Hospital and the Affiliated Cancer Hospital of Xiangya School of Medicine, Central South University, Changsha, Hunan China; 6 The Reproductive Medicine Center, The Third Affiliated Hospital of ShenZhen University, Shenzhen Luohu Hospital Group, Shenzhen, China

**Keywords:** Cancer therapeutic resistance, Autophagy

## Abstract

Drug resistance and recurrence are still the bottlenecks in the clinical treatment of ovarian cancer (OC), seriously affecting patients’ prognosis. Therefore, it is an urgent challenge for OC to be overcome towards precision therapy by studying the mechanism of OC drug resistance, finding new drug resistance targets and developing new effective treatment strategies. In this study, we found that lncRNA LOC730101 played an essential role in attenuating drug resistance in OC. LOC730101 was significantly down-regulated in platinum-resistant ovarian cancer tissues, and ectopic overexpression of LOC730101 substantially increased chemotherapy-induced apoptosis. Mechanistically, LOC730101 specifically binds to BECN1 and inhibits the formation of autophagosome BECN1/VPS34 by reducing phosphorylation of BECN1, thereby inhibiting autophagy and promoting drug sensitivity in ovarian cancer cells following treatment with cisplatin and PARP inhibitors. Moreover, LOC730101 inhibits the expression and activity of RNF168 via p62, which in turn affects H2A ubiquitination-mediated DNA damage repair and promotes drug sensitivity in ovarian cancer cells. Our findings demonstrated that LOC730101 played an important role in regulating the formation of the autophagic complex and that inhibition of autophagy significantly enhances the drug sensitivity of OC. And LOC730101 may be used as a prognostic marker to predict the sensitivity of OC to platinum and PARP inhibitors.

## Introduction

Ovarian cancer (OC) is the third most common gynecological malignancy with the highest mortality rate and is characterized by complicated histology, high malignancy and poor survival prognosis [1]. According to the World Health Organization’s International Agency for Research on Cancer, in 2020, there have 314,000 new cases of ovarian cancer and 207,000 deaths worldwide [2]. Epithelial ovarian cancer (EOC) is the most common in women of all races, accounting for 90% of all ovarian cancer cases. Among them, high-grade serous ovarian cancer (HGSOC) accounts for 70% of all EOC and is the most common histological subtype of EOC [3]. The first-line chemotherapy recommended by NCCN for ovarian cancer is platinum-based with targeted therapies such as PARP inhibitors and angiogenesis inhibitors (e.g., bevacizumab) as maintenance anti-tumor therapy [4], while immunotherapies such as CAR-T [5] therapy, immune checkpoint inhibitors [6] and autologous tumor cell vaccines [7] are still in various clinical trials [8]. The first-line chemotherapy drugs for ovarian epithelial cancer, platinum combined with taxane, have a progression-free survival rate of more than 80%, and more than half of them achieve complete tumor remission. Therefore, chemotherapy is still the first choice for ovarian cancer treatment, but even if complete remission is achieved for the first time, there are still 50–70% of patients develop drug resistance and relapse, and the issues of drug resistance and relapse remain a challenge for clinical treatment.

Targeted therapies for ovarian cancer have made rapid progress in recent years, with the combination of the anti-angiogenic drug bevacizumab improving survival benefits, particularly with PARP inhibitors achieving breakthrough results in patients with advanced disease [9, 10]. As their use becomes more widespread, resistance to PARP inhibitors has become a challenge for patients [11, 12]. Our previous studies have found a direct correlation between platinum sensitivity and PARP inhibitor response, possibly because platinum is a DNA damaging agent that leads to DNA cross-linking, which can partially lead to a decrease in the number of patients treated with PARP inhibitors. This may be because platinum is a DNA damaging agent that causes DNA cross-linking, which is partially repaired by the HR pathway, and PARP inhibitors also inhibit DNA repair in tumor cells.

In summary, the combination of platinum-based, PARP inhibitors and anti-angiogenic therapy has achieved breakthrough efficacy, but drug resistance and recurrence are still bottlenecks in ovarian cancer treatment, and patients are in urgent need of new treatment options that can effectively prolong the interval between platinum-containing chemotherapy and delay the recurrence of ovarian cancer. The development of new drug-resistant targets and new effective therapeutic strategies for ovarian cancer is a pressing challenge for the advancement of precision therapy. The development of new drug-resistant targets and effective therapeutic strategies is therefore a pressing challenge for ovarian cancer to move towards precision treatment.

Autophagy is a complex catalytic process in which excess proteins and subcellular lysosomal degradation of cellular components in the body [13]. Autophagy plays a protective role in tumor therapy [14, 15], leading to treatment tolerance or affecting clinical prognosis. In some of the tumors which are the most difficult to treat autophagy induces resistance in tumor cells to a variety of standard chemotherapeutic agents, e.g., autophagy is responsible for resistance to the cytotoxic drug paclitaxel in ovarian cancer [16], and the resistance of ovarian and oesophageal cancers to cisplatin chemotherapy has been shown to be induced by autophagy [17, 18]. As more and more reports reveal the link between autophagy and drug resistance, autophagy will become a very promising target for malignancy treatment [15]. Autophagy can be prevented by multi-stage targeted inhibition.

Studies have shown that DNA damage caused by various endogenous or exogenous factors that are not repaired in a timely manner will lead to apoptosis, and that DNA damage response plays an important role in the chemotherapy resistance process. Enhanced DNA damage repair is an important molecular basis for drug resistance. The enhanced ability to repair DNA damage is an important molecular basis for drug resistance. Autophagy has been reported to affect DNA damage repair caused by radiotherapy [19]. P62 is the first selective autophagy receptor protein with a UBA domain that binds ubiquitinylated substrate proteins and a LIR domain that binds LC3 [20], p62 protein binds ubiquitinated substrates and autophagic vesicles through the UBA and LIR structural domains, respectively, and transports the substrate to be degraded to the autophagic lysosomal system. When autophagy is not sufficient, it leads to a build-up of p62, which inhibits ubiquitination of histone H2A by RNF168, ultimately affecting the recruitment of RAD51, BRCA1, etc. at sites of damage and preventing DNA repair [20]. Autophagy also ensures the normal regulation of the DNA repair pathway by RNF168 through degradation of the deubiquitinating enzyme USP14 [21].

Long non coding RNAs (LncRNAs) are a family of transcripts greater than 200nt in length that do not encode proteins or have low coding potential [22]. LncRNAs can regulate the response of target cells to drugs by affecting metabolism, apoptosis, autophagy, and the immune microenvironment through the regulation of gene transcription, splicing and other epigenetic processes. LncRNA H19, UCA1, MALAT1, etc. can affect drug sensitivity by participating in drug efflux function [23]. NEAT1, OR3A4, HOTTIP, etc. can play a role in regulating drug sensitivity by regulating the cell cycle [23]. However, the functions and underlying mechanisms of lncRNAs in regulating chemoresistance of OC are largely unknown. Therefore, identifying lncRNAs that possess regulatory roles in chemosensitivity and investigating the underlying mechanisms might provide novel therapeutic targets for OC treatment.

In our present study, we found that lncRNA LOC730101 played an essential role in attenuating chemoresistance in OC. LOC730101 was significantly down-regulated in platinum chemoresistant ovarian cancer and overexpression of LOC730101 greatly increased apoptosis induced by platinum chemotherapy and PARP inhibitors. Mechanistically, high expression of LOC730101 reduces the phosphorylation of BECN1, which in turn inhibits the dissociation of the BECN1-Bcl2 complex, reduces the formation of autophagosomal BECN1-VPS34 and the accumulation of the autophagic substrate p62, which reduces the expression of RNF168 and the p62-bound RNF168 cannot be released to restore its E3 ligase activity, which in turn inhibits histone H2A ubiquitylation, which prevents the recruitment of DNA damage factors such as BRCA1, RAD51 and RAP80 to play a role in DNA damage repair, prevents the repair of DNA damage in ovarian cancer cells, and thus increases the sensitivity of ovarian cancer cells to drugs. Our findings suggest that LOC730101 plays an important role in regulating autophagy and DNA damage repair, and that inhibition of autophagy significantly improves drug sensitivity in ovarian cancer.

## Methods and materials

### Cell lines and cell culture

Human HEK-293T cells, the human OC cell lines A2780, OVCAR3, SKOV3, and CAOV3, and the human normal ovarian epithelial cell line IOSE80 were purchased from American Type Culture Collection (ATCC, USA). For the culture of HEK-293T and A2780 cells, high glucose DMEM (Gibco, USA) supplemented with 10% fetal bovine serum (Gibco, USA), 100 U/ml penicillin (BI, Israel) and 100 μg/ml streptomycin (BI, Israel) were used. OVCAR3, SKOV3, and CAOV3, and the human normal ovarian epithelial cell line IOSE80 cells were maintained in RPMI-1640 (Gibco, USA) supplemented with 10% fetal bovine serum (Gibco, USA), 100 U/ml penicillin (BI, Israel) and 100 μg/ml streptomycin (BI, Israel). The cells were digested with trypsin EDTA solution A (BI, Israel) and cryopreserved with FBS containing 10% DMSO (Sigma-Aldrich, USA).

### Ovarian cancer tissues

A total of eight cases of high-grade plasma ovarian cancer tissues were collected in the first stage, including cancer tissues from five platinum-resistant patients with ovarian cancer and three platinum-sensitive patients with ovarian cancer. The diagnostic criteria for platinum resistance and platinum sensitivity were referred to the Guidelines for the Diagnosis and Treatment of Ovarian Malignancies (2021 edition) [24]. Tissue samples from these eight cases of high-grade plasma ovarian cancer were collected at Hunan Cancer Hospital, and the study was conducted with the consent of ovarian cancer patients, all of whom had signed an informed consent form. All clinical samples were collected with the informed consent of OC patients.

### Organoid culture of ovarian cancer

A group of OC patient samples were collected from patients treated in Hunan Cancer Hospital during the period from December 2021 to July 2022. The short-term organoid culture method of HGSC is as follows: The ovarian cancer tissue obtained during surgery is cut into 2 mm small pieces, and the tissue homogenate is transferred into type II collagenase with a final concentration of 2.5 mg/ml (Life Technologies; #17101015) in the basal medium. Leave at 37 °C for 30 min, shaking every 5 min. Then the homogenate was diluted 1:1 with the base medium and filtered through a 70 μm filter (Falcon; 352350). The cell suspension was centrifuged at 1000 rpm/min to obtain cell precipitation. Red blood cell lysate (BioLegend; 420301) rinse 2 to 3 times, and finally rinse once with base medium. The obtained cell and matrix gel (Corning; CB-40230C) mixture, the final concentration of matrix glue was 75%, and every 10 μl cells and matrix glue mixture contained about 10,000 cells. Then the 15 μl mixture was quickly seeded into the 48-well plate. After the mixture was cured, 250 μl medium was added to each well. (Medium was DMEM/F12 (Thermo Fisher; 12634028), add 1% penicillin-streptomycin, 1×Glutamax; Life Technologies; 35050061), 1% HEPES (Life Technologies; 15630080), 100 ng/ml R-spondin 1 (PeproTech; 120-38), 100 ng/ml Noggin (PeproTech; 120-10 C), 50 ng/ml EGF (PeproTech; 100-15), 10 ng/ml FGF-10 (PeproTech; 100-26), 10 ng/ml FGF2 (PeproTech; 100-18B), 1×B27 (Life Technologies; 17504044), 10 mmol/l nicotinamide (Sigma-Aldrich; N0636), 1.25 mmol/l N-acetylcysteine (N-acetylcysteine; Sigma-Aldrich; A9165), 1 μmol/l prostaglandin E2 (R&D Systems; 2296), 10 μmol/l SB202190 (Sigma-Aldrich; S7076) and 500 nmol/l A83-01 (Sigma-Aldrich; SML0788). Y-27632 Dihydrochloride (AbMole Bioscience; M1817) at a concentration of 10 μmol/l in early organoid culture, but not required in subsequent culture. Replace the medium every 2–4 days, generally 7–10 days of culture can be used for follow-up experiments, 10–14 days of passage. During passage, the matrix gel containing organoids was incubated with TrypLE Express (Thermo Fisher Scientific, 12604013) for 30 min, and then the cell suspension was centrifuged at 4 °C at 1500 rpm for 5 min. The resulting cell precipitates were resuscitated in a cold matrix gel and reseeded into the culture plate.

### Stable cell line establishment

The plasmid of pLV-puro-LOC730101 (10 μg) or pLKO.1-puro-shLOC730101 (10 μg) or corresponding control vector were co-transfected with the two packaging vectors (7.5 μg pSPAX2 and 7.5 μg pMD2G), respectively, into 293FT cells for 48 h to generate lentivirus. Supernatant medium containing the target lentivirus was collected and then OC cells were infected with lentivirus mixed with Polybrene (Santa Cruz Biotechnology, CA, USA). The construct sequences of the stable expression vectors are shown in Table S1.

### Cell transfection

One day before transfection, OC cells in good logarithmic growth phase were trypsinized, prepared into cell suspension and inoculated into 6-well plates to ensure a cell density of 40%–50%. Transfection was carried out according to the instructions for Hipperfect transfection reagent. After 48 h of transfection the RNA and protein of the transfected cells were extracted to verify the interference efficiency of siRNA. All siRNA sequences involved in the text are shown in Table S2.

### Immunoblotting assay

Cell lysates were made with RIPA mixed buffer containing protease inhibitors and phosphatase inhibitors. Total cell extracts were separated by SDS-PAGE and transferred to PVDF Membrane (Milipore, Germany) and incubated with primary antibodies: anti-BECN1 (11306-1-AP, Proteintech) anti-Bcl2 (14600-1-AP, Proteintech), anti-LC3 (12789-1-AP, Proteintech), anti-RNF168 (21393-1-AP, Proteintech), anti- p62/SQSTM1 (18420-1-AP, Proteintech) and β-Actin (66009-1-Ig, Proteintech) as the internal reference. Membranes were then incubated with HRP-conjugated anti-mouse or anti-rabbit IgG antibodies, depending on the source of the primary antibody. The membranes were then imaged with a ChemiDoc MP system (Bio-Rad, USA).

### RNA immunoprecipitation (RIP)

Take 30 µl of Protein-A/G (Santa Cruz Biotechnology, USA), agarose beads with 3–5 µg of each of the target antibody, IgG, and incubate in 300 µl of GLB+ buffer at room temperature with slow rotation for 2 h. Wash the antibody-bound agarose beads with GLB+ twice, and place the bound agarose beads on ice for spare time. Prepare fresh cell IP lysate (1 ml IP lysate with 10 µl 1 M PMSF, 10 µl 0.1 M DTT, 10 µl 100×Cocktail, and 10 µl RNase inhibitor). Wash the adherent ovarian cancer cells three times with pre-cooled PBS, add the prepared IP lysate to cover the surface of the cells in a homogeneous manner, and lyses were performed on ice for 1 min. Blow the lysate and cells repeatedly to fully lyse the adherent cells, transfer the lysed cell suspension to a new EP tube, and after 10 min of lysis on ice, centrifuge the cells at 13,000 rpm for 10 min at 4 °C, and aspirate the protein supernatant into a new EP tube. 50 µl of protein supernatant was retained as Input and 700 µl of TRIzol lysate was added for total RNA extraction. The remaining protein supernatant was proportionally added to the yeast tRNA (4 µl of 10 mg/ml of yeast tRNA per 1 ml of supernatant) and divided into two portions, which were added to the antibody agarose bead conjugate tubes and conjugated by slow spinning at 4 °C for 5 h. The antibody-protein conjugated agarose beads were washed rapidly twice, centrifuged at 4 °C for 1 min at 3000 rpm, and washed slowly for three times, then washed slowly for two hours at 4 °C for three minutes. The supernatant was removed by centrifugation at 4 °C and 3000 rpm for 1 min at 4 °C. 700 µl of TRI-conjugated beads were added to each tube. Add 700 µl TRIzol lysate per tube and store at −80 °C or extract RNA according to the RNA Extraction Kit RNeasy® Mini instructions. The fold enrichment of precipitated LOC730101 RNA was examined by RT-qPCR.

### RNA pull-down assay

Full length LOC730101 RNA (LOC730101(+)) and LOC730101-antisense RNA (LOC730101(-)) were transcribed in vitro from pcDNA3.1-LOC730101(+) and pcDNA3.1- LOC730101(-) and labeled with biotin by the Biotin RNA Labeling Mix (Sigma, USA) and T7 RNA polymerase (Sigma, USA). The biotinlabeled RNAs (3 μg) was heat-excited at 90 °C for 2 min and immediately placed on ice for 2 min. Twice the volume of RNA structure buffer was added and left to fold for 20 min at room temperature. the folded RNA was mixed with the protein samples and incubated in a rotary mixer in a slow rotary incubator at room temperature for 1.5 h. The RIP buffer was washed with Pierce Streptavidin Agarose beads (Thermo Fisher Scientific Inc., Waltham, MA, USA) twice. Add 50 µl of washed magnetic beads to each sample and place on a rotary mixer for 1 h of slow rotary incubation at room temperature. Gently wash the bound beads 5 times with RIP buffer, resuspend the beads with 50 µl of RIP buffer, and finally add 10 µl of SDS buffer and denature in boiling water for 10 min. The retrieved proteins were subjected to SDS-PAGE gel electrophoresis and the protein band of the LOC730101 group was subjected to silver staining, and differential protein bands were identified using high resolution mass spectrometry. The identified proteins were examined using regular Western Blot assay.

### Xenografted tumor models

Thirty BALB/c nude mice were purchased through the Department of Experimental Zoology, Central South University. The basic information of the mice is: SPF class, female, age 3 weeks old, weight 16–18 g. The mice are housed in the barrier system of the Department of Zoology, Central South University. After one week of acclimatization in foster care, OVCAR3 Vector cells (15 in the control group) and OVCAR3 OE-LOC730101 cells (15 in the experimental group) were injected subcutaneously. The cell injection volume was 2 × 10^6^ each and the cell injection suspension was 100 µl per nude mouse. The injections were given for about 10 days and the tumors were treated by dosing when they had grown to the size of a green bean. The control and experimental groups were further randomly divided into 3 groups (5 mice in each group) and treated with saline, cisplatin and niraparib respectively. The tumor growth of the mice was observed and the length and width of the tumors were measured every other day.

After administration, the mice were euthanised and dissected to obtain tumor tissue samples from each mouse and the tumors were weighed. The volume of the tumor was calculated by the formula (V = 1/2 × L × W^2^) and the tumor tissue was used for paraffin embedding to produce paraffin sections for subsequent H&E staining, in situ hybridization and immunohistochemistry.

### High throughput transcriptome sequencing

Cancer tissue samples from 8 patients with platinum-sensitive and platinum-resistant high-grade serous ovarian cancer were sent to BGI for high-throughput transcriptome sequencing, and subsequent differential expression analysis was performed on the BGI Multi-genome system platform (https://biosys.bgi.com). The company first qualifies and filters the raw sequencing data to remove low-quality read segments and splices. The clean data is then compared to the reference genome. Then, the gene expression level was quantified by comparing the results. Then we conducted differential expression analysis on the BGI Multi-genome system platform. ‌The identification criteria for differential genes we set were |log2 Fold Change | >1, FPKM > 1 and *P* < 0.001.

### Statistical analysis

All date statistics for qRT-PCR, CCK-8, and mouse xenograft tumor models were expressed as mean ± SEM. The clinicopathological features of ovarian cancer tissue samples were analyzed by t-test. Statistical analysis was calculated with GraphPad Prism 7.0. The differences between groups were analyzed using the t-test or two-way ANOVA. A value of *P* < 0.05 (*), <0.01 (**) or <0.001 (***) was considered statistically significant.

## Results

### LOC730101 is up-regulated in platinum-sensitive OC tissues

**Fig. 1:**
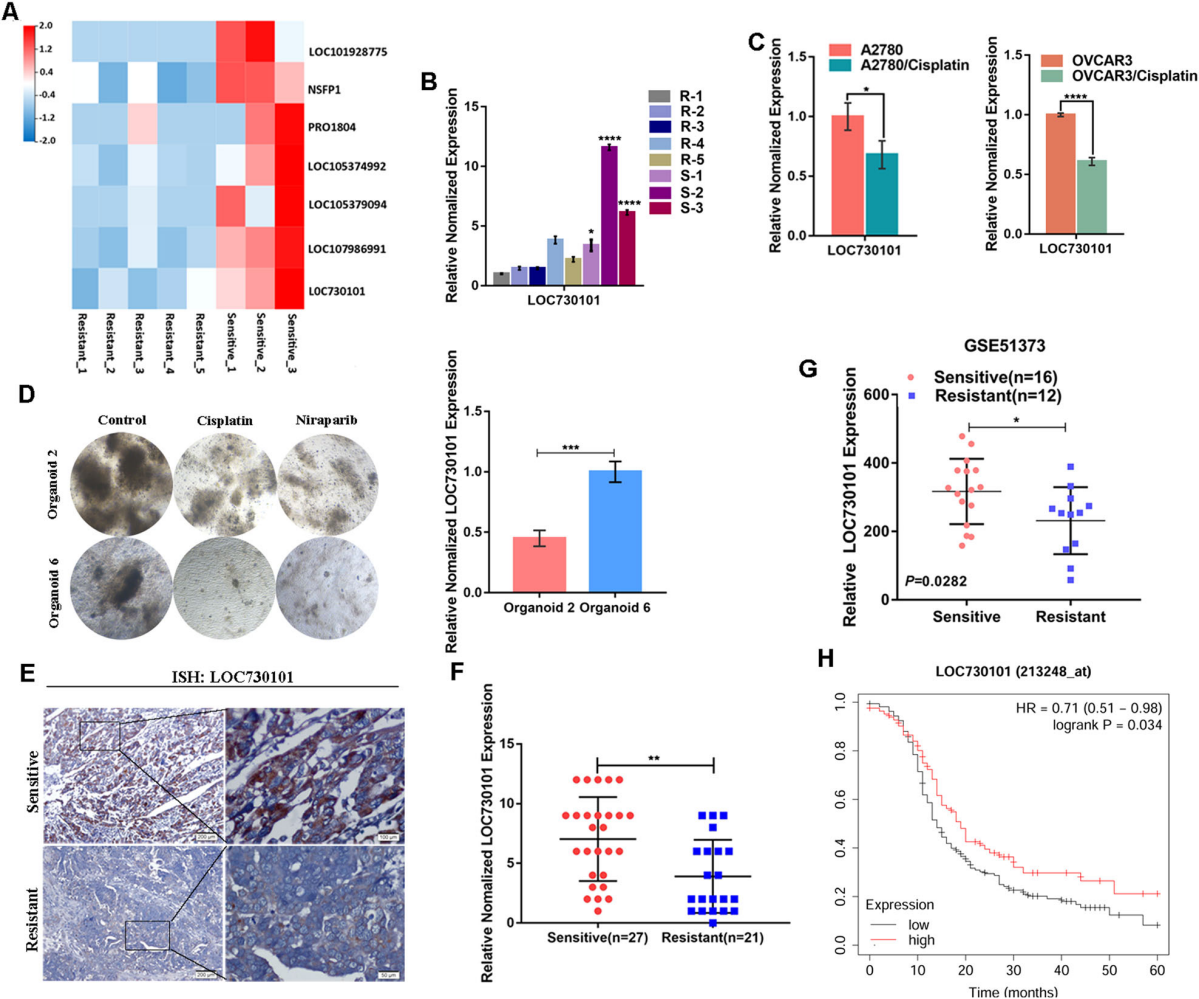
LOC730101 is highly expressed in platinum sensitive ovarian cancer and is positively related to the prognosis of patients. **A** Heat map analysis of high-throughput transcriptome sequencing results showed that LOC730101 was significantly overexpressed in platinum sensitive ovarian cancer tissues, log2 fold change>1, FPKM > 1, *p* < 0.001. **B** The expression of LOC730101 in platinum sensitive ovarian cancer (n = 3) and platinum resistant ovarian cancer (n = 5) was detected by qPCR. Actin was the internal reference. **C** The expression of LOC730101 in A2780 and A2780/Cisplatin (OVCAR3 and OVCAR3/Cisplatin) ovarian cancer cell lines was detected by qPCR. Actin was the internal reference. t-test, *, *p* < 0.05, ****, *p* < 0.0001. **D** Morphogram of an ovarian cancer-derived organoid after cisplatin and niraparib treatment. **E** In situ hybridization assay to detect the expression of LOC730101 in platinum-sensitive ovarian cancer tissues (n = 27) and platinum-resistant ovarian cancer tissues (n = 21). Scale bars, 100 µm and 20 µm. **F** Statistical plots of LOC730101 in platinum-sensitive ovarian cancer tissues (n = 27) and platinum-resistant ovarian cancer tissues (n = 21) for scoring in situ hybridization assay. t-test, **, *p* < 0.01. **G** GSE51373 data analysis results showed that the expression of LOC730101 in platinum sensitive tissues (n = 16) of ovarian cancer was higher than that in platinum resistant tissues (n = 12), t-test, *p* = 0.0282. **H** The survival curve showed that the expression of LOC730101 was positively correlated with the progression free survival of ovarian cancer patients, t-test, *p* = 0.034.

We used high-throughput transcriptome sequencing to compare the differentially expressed lncRNAs in cancer tissue samples from platinum-sensitive (n = 5) and platinum-resistant (n = 3) high-grade plasma ovarian cancer patients. Heat map analysis of the top 7 significantly up-regulated lncRNAs in the platinum-sensitive group showed that lncRNA LOC730101 was significantly highly expressed in platinum-sensitive tissues of ovarian cancer (Fig. 1A), and LOC730101 was identified and verified by qPCR to be indeed highly expressed in platinum-sensitive tissues of ovarian cancer (Fig. 1B). The qPCR assay showed decreased expression of LOC730101 in both A2780/Cisplatin and OVCAR3/Cisplatin cell lines in the cisplatin-resistant group compared to the control cisplatin-sensitive group (Fig. 1C). We cultured ovarian cancer tissue-derived organoids and performed drug sensitivity assays, which showed that organoids with high LOC730101 expression were more sensitive to cisplatin and niraparib than those with LOC730101 low expression (Fig. 1D and Fig. S1). And we analyzed the expression of LOC730101 in 27 cases of platinum-sensitive OC tissues and 21 cases of platinum-resistant OC tissues. The results showed that LOC730101 were highly expressed in platinum-sensitive OC compared with platinum-resistant OC tissues (Fig. 1E, F). Analysis of the GSE51373 ovarian cancer data downloaded from the GEO database revealed that LOC730101 expression was up-regulated in platinum-sensitive tissues of ovarian cancer compared to platinum-resistant tissues, and analysis of the TCGA ovarian cancer database revealed that high expression of LOC730101 was positively associated with progression-free survival in ovarian cancer patients (Fig. 1G, H). It is suggested that LOC730101 was highly expressed in platinum-sensitive tissues of ovarian cancer and positively correlated with patient prognosis.

### LOC730101 regulation of drug sensitivity in ovarian cancer cells

LOC730101 (Ensembl ID: ENSG00000216775, Gene ID: AL109918.1) is located on human chromosome 6p12.2 and contains 2 exons with a transcript length of 3784 bp (Fig. S2A), which was predicted via the online CAPT website (http://lilab.research.bcm.edu/cpat/) predicted to be non-coding and the size of the longest open reading frame was 285 bp (Fig. S2B), its function and mechanism of action in ovarian cancer have not been reported. In order to further clarify the function and mechanism of action of LOC730101 in ovarian cancer cells, the expression of LOC730101 in normal ovarian epithelial cells and various ovarian cancer cell lines was firstly examined (Fig. S3). Based on the expression status, we selected ovarian cancer A2780 and OVCAR3 cells as the subjects and further explored the functions and mechanisms of LOC730101 in ovarian cancer cells by constructing ovarian cancer cell lines with stable overexpression and stable knockdown of LOC730101.

**Fig. 2:**
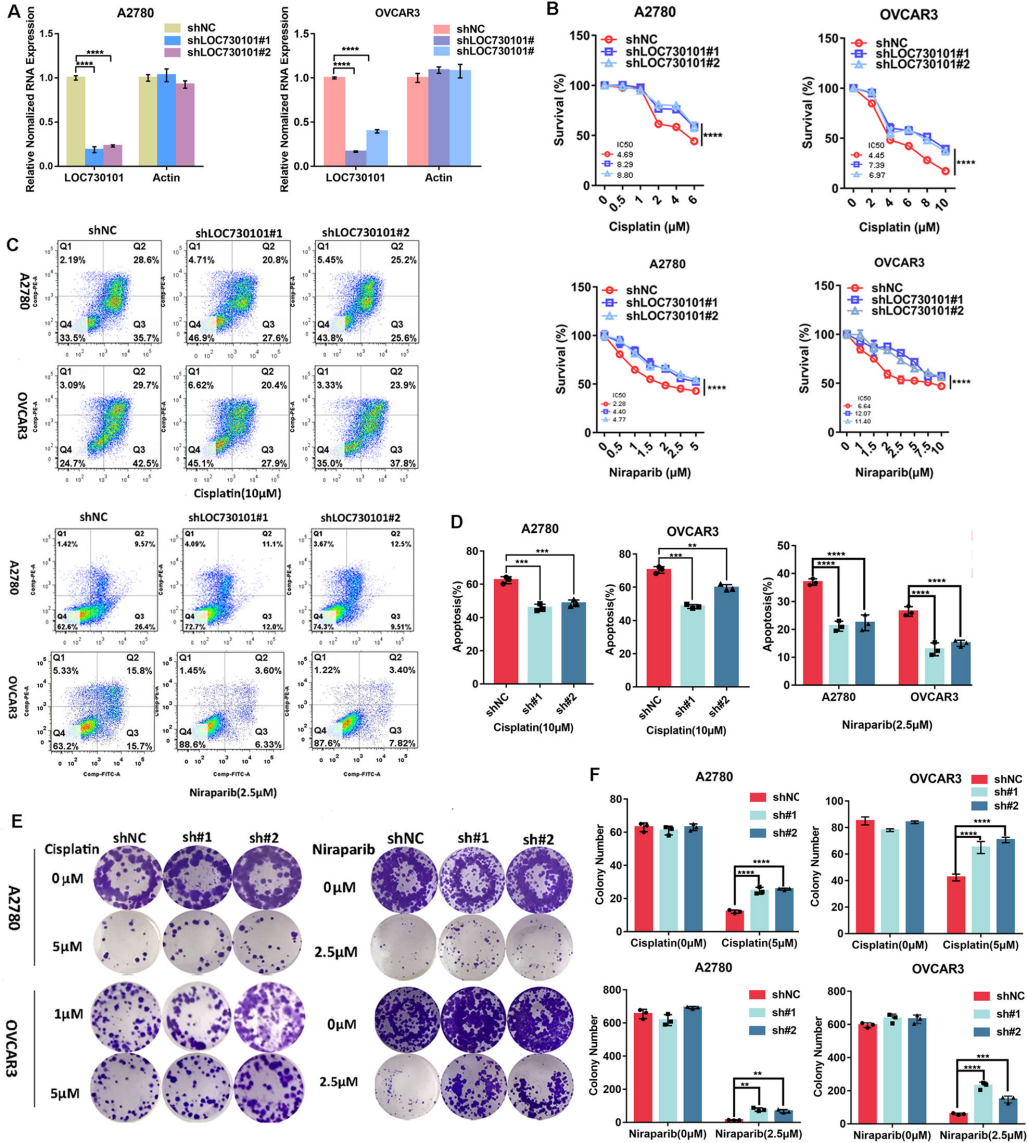
Knocking down the expression of LOC730101 inhibits the drug sensitivity of ovarian cancer cells. **A** qPCR was used to detect the expression of LOC730101 in A2780 shNC and A2780 shLOC730101#1/#2 (OVCAR3 shNC and OVCAR3 shLOC730101 #1/#2) ovarian cancer cells. Actin was an internal reference, t-test, ****, *p* < 0.0001. **B** The ovarian cancer cells were treated with gradient concentrations of cisplatin or niraparib for 72 h, respectively. CCK8 detected the cell activity and IC_50_ value of A2780 shNC and A2780 shLOC730101 #1/# 2 (OVCAR3 shNC and OVCAR3 shLOC730101 #1/#2) ovarian cancer cells, t-test, ****, *p* < 0.0001. **C** Ovarian cancer cells were treated with 10 µM cisplatin or 2.5 µM niraparib for 72 h. The apoptosis rates of A2780 shNC and A2780 shLOC730101 #1/#2 (OVCAR3 shNC and OVCAR3 shLOC730101 #1/#2) ovarian cancer cells were detected by flow cytometry. **D** Statistical chart of apoptosis rate detected by flow cytometry. two-way ANOVA, **, *p* < 0.01, ***, *p* < 0.001. **E** Ovarian cancer cells were treated with cisplatin or niraparib for 72 h, and then replaced with normal complete medium for 10 days. The proliferation of A2780 shNC and A2780 shLOC730101 #1/# 2 (OVCAR3 shNC and OVCAR3 shLOC730101 #1/#2) ovarian cancer cells were detected by clonogenic assay. **F** Statistical chart of the results of clone formation experiment. two-way ANOVA, ***, *p* < 0.0001.

The lentiviral expression system was used to construct ovarian cancer cell lines with stable knockdown of LOC730101, qPCR results showed that that its inhibition rate is 60–70% (Fig. 2A). After treatment with cisplatin, it was found that the survival ratio of ovarian cancer cells with lower level of LOC730101 was significantly higher than that of the control group (Fig. 2B); the number of colonies of ovarian cancer cells with low expressing LOC730101 was increased compared with that of the control group (Fig. 2E, F); the apoptosis rate of the ovarian cancer cells with low expressing LOC730101 was significantly lower than that of the control group (Fig. 2C, D). It indicated that knockdown of LOC730101 inhibited the sensitivity of ovarian cancer cells to cisplatin drug.

Targeted therapies for ovarian cancer have gained rapid progress in recent years, especially PARP inhibitors, which have brought new benefits to patients with advanced ovarian cancer [9, 25], but as their use becomes more widespread, resistance to PARP inhibitors has become a challenge for ovarian cancer patients. A direct correlation between platinum sensitivity and PARP inhibitor response has also been found, so in this study we also explored the effect of LOC730101 on the efficacy of the PARP inhibitor niraparib. When ovarian cancer cells were treated with the PARP inhibitor niraparib, the survival of ovarian cancer cells with low expressing LOC730101 was significantly higher than that of the control group (Fig. 2B); the number of colonies formation was increased in ovarian cancer cells with low expressing LOC730101 compared to the control group (Fig. 2E, F); the apoptosis rate of ovarian cancer cells with low expressing LOC730101 was significantly lower than that of the control group (Fig. 2C, D). This indicates that knockdown of LOC730101 also inhibited the sensitivity of ovarian cancer cells to PARP inhibitors.

**Fig. 3:**
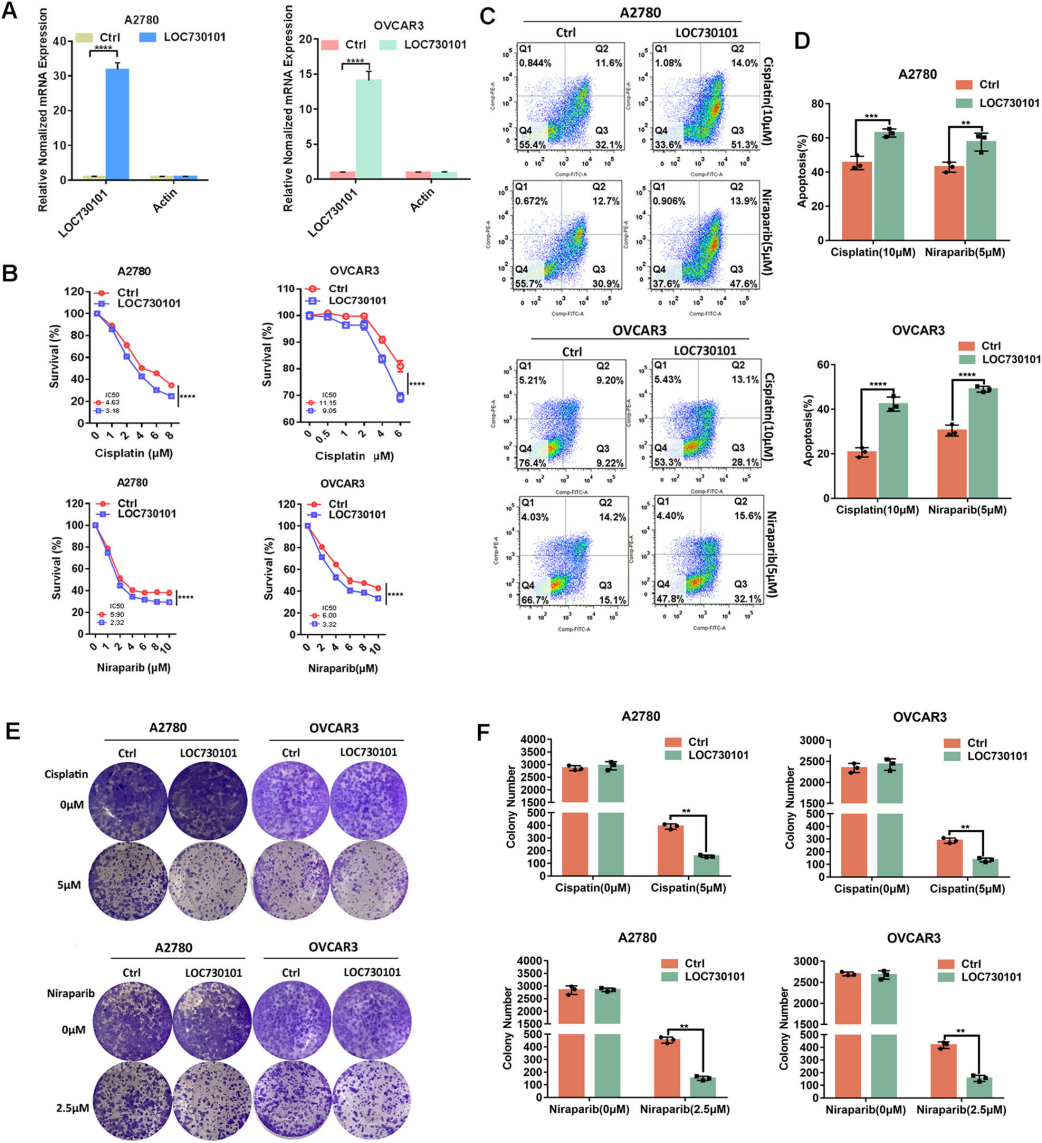
Overexpression of LOC730101 promotes drug sensitivity of ovarian cancer cells. **A** qPCR was used to detect the expression of LOC730101 in A2780 Ctrl and A2780 OE-LOC730101 (OVCAR3 Ctrl and OVCAR3 OE-LOC730101) ovarian cancer cells. Actin was an internal reference, t-test, ****, *p* < 0.0001. **B** The ovarian cancer cells were treated with gradient concentrations of cisplatin or niraparib for 72 h, respectively. CCK8 detected the cell activity and IC_50_ value of A2780 Ctrl and A2780 OE-LOC730101 (OVCAR3 Ctrl and OVCAR3 OE-LOC730101) ovarian cancer cells, two-way ANOVA, ****, *p* < 0.0001. **C** Ovarian cancer cells were treated with 10 µM cisplatin or 2.5 µM niraparib for 72 h. The apoptosis rates of A2780 Ctrl and A2780 OE-LOC730101 (OVCAR3 Ctrl and OVCAR3 OE-LOC730101) ovarian cancer cells were detected by flow cytometry. **D** Statistical chart of apoptosis rate detected by flow cytometry. two-way ANOVA, **, *p* < 0.01, ***, *p* < 0.001. **E** Ovarian cancer cells were treated with cisplatin or niraparib for 72 h, and then replaced with normal complete medium for 10 days. The proliferation of A2780 Ctrl and A2780 OE-LOC730101 (OVCAR3 Ctrl and OVCAR3 OE-LOC730101) ovarian cancer cells were detected by clonogenic assay. **F** Statistical chart of the results of clone formation experiment. two-way ANOVA, ***, *p* < 0.001.

We also constructed A2780 and OVCAR3 ovarian cancer cell lines stably overexpressing LOC730101. qPCR results indicated that ovarian cancer cell lines stably overexpressing LOC730101 were successfully constructed (Fig. 3A). After treatment of ovarian cancer cells with cisplatin and niraparib, respectively, it was found that the survival of ovarian cancer cells with LOC730101 overexpression was significantly lower than that of the control group (Fig. 3B); the number of colonies of ovarian cancer cells with LOC730101 overexpression was significantly reduced compared with that of the control group (Fig. 3E, F); the apoptosis rate of ovarian cancer cells with LOC730101 overexpression was significantly more than that of the control group (Fig. 3C, D). This indicates that the sensitivity of ovarian cancer cells with high expression of LOC730101 to both cisplatin and niraparib was enhanced.

### LOC730101 directly interacted with autophagy key protein BECN1

**Fig. 4:**
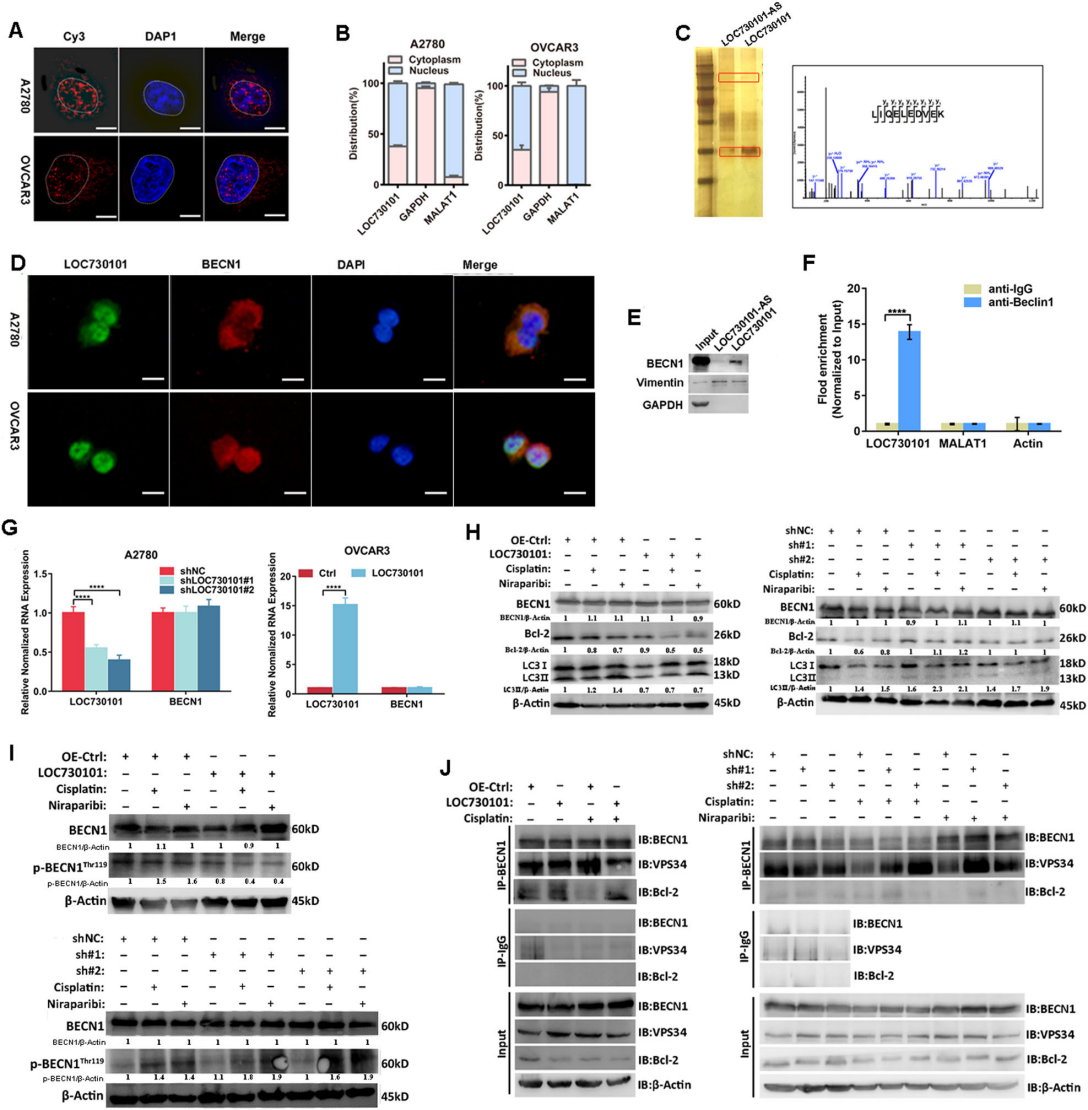
LOC730101 binds specifically to the key autophagy protein BECN1 and regulates autophagy in ovarian cancer cells. **A** Fluorescence in situ hybridization was used to detect the expression and location of LOC730101 (red) in ovarian cancer A2780 and OVCAR3 cells, and DAPI (blue) labeled the nucleus. Scale, 10 µm. **B** The expression of LOC730101 in A2780 and OVCAR3 cells was detected by RNA nuclear and cytoplasmic separation test combined with qPCR, MALAT1 was the nuclear reference and GAPDH was the cytoplasmic reference. **C** RNA pull-down combined with silver staining of immunoblotting showed that there were differential protein bands between the sense chain and antisense chain of LOC730101. Mass spectrometry analysis detected the absorption peak map of the peptide segment of the key autophagy protein BECN1. **D** The expression and localization of LOC730101 (green) and BECN1 (red) in A2780 and OVCAR3 ovarian cancer cells were detected by fluorescence in situ hybridization (FISH) combined with immunofluorescence assay. DAPI (blue) labeled the nucleus. Scale, 10 µm. **E** RNA pull-down combined with Western Blot was used to detect the expression of BECN1 in the complex precipitated by LOC730101. Vimentin was the positive control, and GAPDH was the internal reference. **F** RIP experiment combined with qPCR was used to detect the expression of LOC730101 in the complex precipitated by IgG antibody and BECN1 antibody. MALAT1 was the negative control, and Actin was the internal reference. t-test, ****, *p* < 0.0001. **G** The RNA expressions of LOC730101 and BECN1 in A2780 shNC and A2780 shLOC730101#1/#2 (OVCAR3 Ctrl and OVCAR3 OE-LOC730101) cells were detected by qPCR, Actin was an internal reference. t-test, ****, *p* < 0.0001. **H** Western blot was used to detect the expression of BECN1, Bcl2 and LC3 in control and overexpression LOC730101 (shNC and shLOC730101) ovarian cancer cells. Actin was used as an internal reference. **I** Western blot was used to detect the expression of p-BECN1^Thr119^ in control and overexpression LOC730101 (control and knock down LOC730101) ovarian cancer cells. Actin was used as an internal reference. **J** Ovarian cancer cells were treated with 10 µM cisplatin or 10 µM niraparib for 24 h, Co-IP assay combined with Western Blot was used to detect the expression of BECN1, VPS34 and Bcl2 in Input, IgG antibodies and BECN1 antibody precipitated complexes in control group and overexpression LOC730101 (control and knock down LOC730101) ovarian cancer cells. Actin was used as an internal reference.

We further explore the mechanism of LOC730101 involvement in the regulation of drug sensitivity in ovarian cancer cells. The subcellular localization of lncRNA could suggest the mechanism of its action. The subcellular localization of LOC730101 was determined by fluorescence in situ hybridization assay and RNA nucleoplasm separation assay, and the results showed that LOC730101 in both the nucleus and cytoplasm of ovarian cancer cells (Fig. 4A, B). We consider the mechanism of action of lncRNA binding proteins, we performed RNA pull-down assays, followed by electrophoresis, silver staining and mass spectrometry (MS) analysis. Among the proteins identified by MS, the autophagy key protein BECN1 was of interest to us and was identified as a candidate protein for interaction with LOC730101 (Fig. 4C). Fluorescence in situ hybridization combined with immunofluorescence assays confirmed the spatial co-localization of LOC730101 with BECN1 (Fig. 4D), and RNA pull-down assays combined with Western Blot and RIP assays further confirmed the interaction of LOC730101 with BECN1 (Fig. 4E, F). It is suggested that LOC730101 may play a role in promoting drug sensitivity in ovarian cancer cells by specifically binding to the autophagy key protein BECN1.

### LOC730101 regulates autophagy and apoptosis in ovarian cancer cells

The BECN1 gene is located on human chromosome 17q21 and contains a total of 11 introns and 12 exons [26]. ULK1 kinase induces autophagy by activating VPS34 through phosphorylation of BECN1 [27, 28]. On the one hand, BECN1 promotes autophagy initiation by forming autophagosomes with VPS34 [29], and on the other hand, it can bind to the anti-apoptotic protein Bcl2 when encountered with apoptotic signals, causing Bcl2 to dissociate from the pro-apoptotic protein Bax, thus playing a role in activating apoptosis and inhibiting cellular autophagy [29, 30]. Apoptosis and autophagy play a very important role in maintaining cellular homeostasis and there is a complex relationship between them. The Bcl2/BECN1 complex acts as a “switch” in the development of apoptosis and autophagy, determining whether the cell enters the apoptotic program or initiates the autophagic program.

We therefore hypothesized that LOC730101 might play a role in promoting drug sensitivity in ovarian cancer cells through the regulation of autophagy and apoptosis by BECN1. Q-PCR results showed that neither stable overexpression nor stable knockdown of LOC730101 affected BECN1 RNA expression (Fig. 4G). Western Blot showed that after treatment of ovarian cancer cells with cisplatin and niraparib, respectively, neither stable overexpression nor stable knockdown of LOC730101 affected the protein expression of BECN1, but after drug treatment, overexpression of LOC730101 compared with the control group, the autophagy marker molecule LC3 II/LC3 I ratio was reduced, while the trend was reversed in the knockdown group, and the anti-apoptotic protein Bcl2 was reduced in the overexpression LOC730101 group compared with the control group (Fig. 4H). This indicates that after treatment with drugs, high expression of LOC730101 inhibited the autophagy of ovarian cancer cells and promoted ovarian cancer cell apoptosis.

### LOC730101 inhibits autophagic complex BECN1-VPS34 formation by decreasing BECN1 phosphorylation

Phosphorylation is considered to be a key modification of BECN1, and phosphorylated BECN1 at different sites is closely associated with downstream biological behaviors, e.g., it has been reported that IL-6 regulates autophagy and chemoresistance by promoting BECN1 Y333 phosphorylation [31], and it has also been reported that activated ROCK1 can promote autophagy by binding and phosphorylating BECN1 at Thr119 occurrence [32]. We therefore speculated whether LOC730101 also regulates autophagy by affecting BECN1 phosphorylation. To further verify this, we examined the expression of phosphorylated BECN1 in stably overexpressed and stably knockdown LOC730101 cells before and after drug treatment by Western Blot and found that high expression of LOC730101 could inhibit the expression of p-BECN1^Thr119^ expression, whereas p-BECN1^Thr119^ expression was increased in knockdown LOC730101 ovarian cancer cells (Fig. 4I).

Phosphorylated BECN1 activates VPS34 and induces autophagy [26], and an increase in Thr119 phosphorylated BECN1 promotes the specific dissociation of the BECN1-Bcl2 complex [32], which further binds to VPS34 to form an autophagic complex to promote autophagy initiation. Western Blot assays showed that when ovarian cancer cells were treated with cisplatin or niraparib, respectively, high expression of LOC730101 inhibited the BECN1-VPS34 complexes compared to controls and promotes the formation of BECN1-Bcl2 complex and thus inhibited the formation of BECN1-VPS34 autophagosomes, while knockdown of LOC730101 promoted the specific dissociation of BECN1-Bcl2 complex and thus the formation of BECN1-VPS34 autophagosomes compared with the control group (Fig. 4J). These data suggest that LOC730101 inhibits BECN1 phosphorylation and thus inhibits the formation of autophagosomal BECN1-VPS34 by binding to BECN1.

### LOC730101 specifically associates with BECN1 and inhibits the phosphorylation site of BECN1

**Fig. 5:**
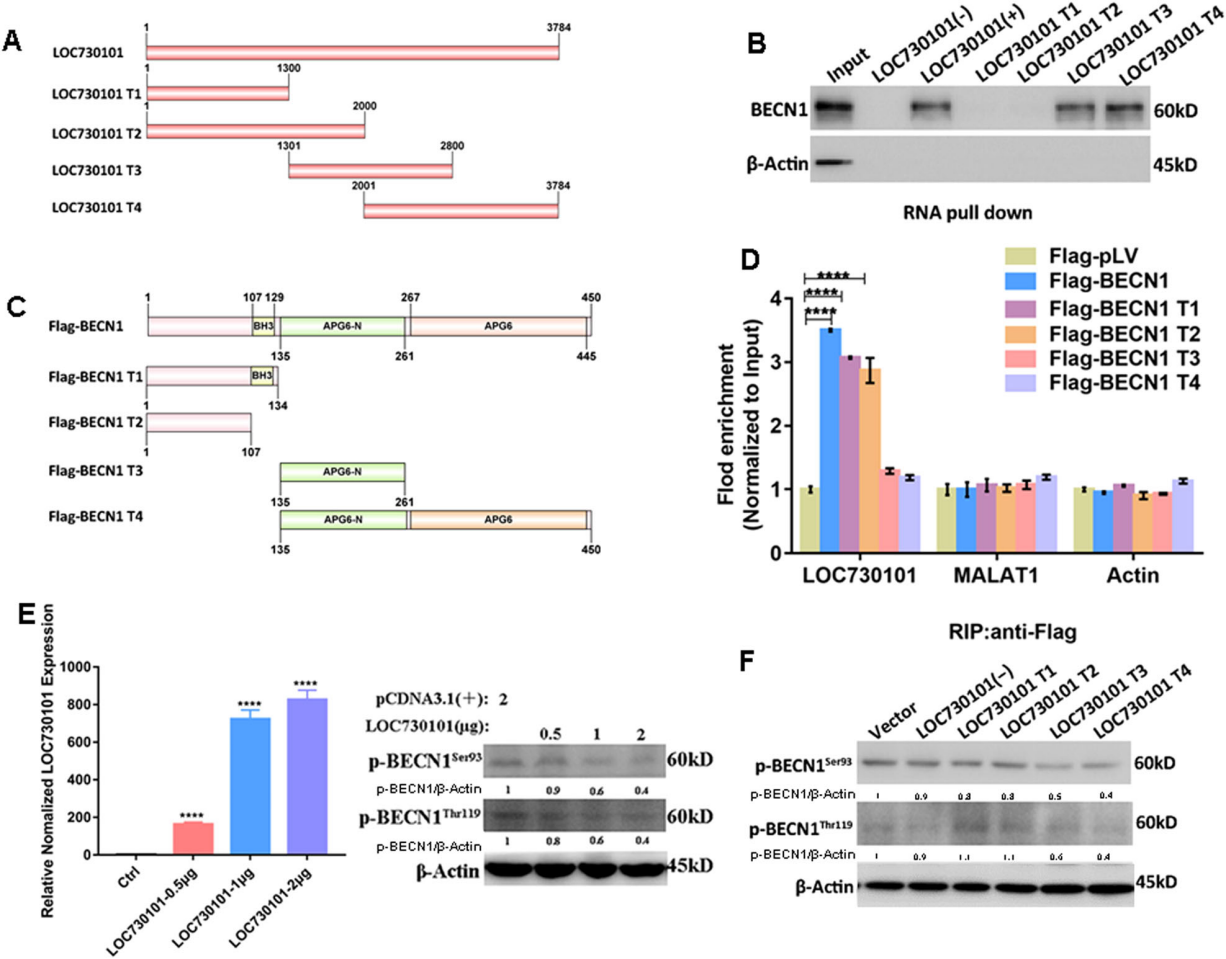
LOC730101 specifically associates with BECN1 and inhibits the phosphorylation site of BECN1. **A** Schematic diagram of LOC730101 truncated mutant. **B** The expression of BECN1 protein was detected by the RNA pull-down test of LOC730101 antisense chain and its deletion mutant combined with Western Blot, β-actin is an internal reference. **C** Schematic diagram of construction of Flag-BECN1 full length and deletion mutants. **D** After HEK293 cells were transfected with Flag-BECN1 full length, Flag-BECN1 T1, Flag-BECN1 T2, Flag-BECN1 T3 and Flag-BECN1 T4 plasmids for 72 h, the RNA expression of LOC730101 was detected by RIP experiment and qPCR. MALAT1 was the negative control, and actin acted as the internal reference, t-test, ****, *p* < 0.0001. **E** After transfection with gradient increased LOC730101 plasmid for 72 h, the expression of p-BECN1^Thr119^ and p-BECN1^Ser93^ protein was detected by Western Blot, β-actin is an internal reference. **F** After the deletion mutant LOC730101 T1, T2, T3 and T4 plasmids were transfected into cells for 72 h, the protein expression of p-BECN1^Thr119^ and p-BECN1^Ser93^ was detected by Western Blot.

To further explore how LOC730101 inhibits BECN1 phosphorylation, we identified the specific RNA sequences where LOC730101 binds to the BECN1 protein. Deletion mutants LOC730101 T1, T2, T3 and T4 of LOC730101 (1-1300nt, 1-2000nt, 1301-2800nt and 2001-3784nt) were constructed based on the RNA sequence of LOC730101 (Figs. 5A and S4A). Next, BECN1 protein expression was detected by RNA pull-down assay combined with Western Blot, which showed that when RNA pull-down was performed using LOC730101 antisense strand, deletion mutant LOC730101 T1 (1-1300nt) and LOC730101 T2 (1-2000nt), BECN1 protein was not detected. In contrast, the BECN1 protein was detected when RNA pull-down was performed using the LOC730101 sensestrand, LOC730101 T3 (1301-2800nt) and LOC730101 T4 (2001-3784nt). It was inferred that LOC730101 specifically binds to the BECN1 protein through its 3′ end region (2001-3784nt) (Fig. 5B).

We constructed Flag-tagged BECN1 full-length and truncated Flag-BECN1 T1 (1-134aa), Flag-BECN1 T2 (1-107aa), Flag-BECN1 T3 (135-261aa) and Flag-BECN1 T4 (135-450aa) plasmids based on the protein structural domain of BECN1, and transfected them into HEK293 cells, where they were successfully expressed by Western Blot (Figs. 5C and S4B). RIP experiments combined with qPCR to detect LOC730101 RNA expression showed that LOC730101 was significantly enriched only when RIP experiments were performed in cells transfected with the Flag-BECN1 full-length and deletion mutants Flag-BECN1 T1 (1-134aa) and Flag-BECN1 T2 (1-107aa). It was inferred that the BECN1 amino acid sequence 1-134aa binds to LOC730101 (Fig. 5D). This region is the N-terminal region of BECN1 and contains multiple phosphorylation sites as well as a Bcl2 homology 3 (BH3) structural domain required for interaction with the Bcl2 protein. We therefore hypothesize that LOC730101 acts to reduce BECN1 phosphorylation by specifically binding to BECN1 and inhibiting its phosphorylation site.

We then further examined the expression of phosphorylated BECN1 by transfecting gradients of LOC730101, which showed that the expression gradients of p-BECN1^Thr119^ and p-BECN1^Ser93^ decreased with increasing LOC730101 (Fig. 5E). Finally, the expression of p-BECN1^Thr119^ and p-BECN1^Ser93^ was detected by Western Blot when transfected with LOC730101 antisense strand and deletion mutants LOC730101 T1, T2, T3 and T4, and the results showed that when transfected with deletion mutants LOC730101 T3 and T4, the expression of p-BECN1^Thr119^ and p-BECN1^Ser93^ expression was reduced (Fig. 5F). It is further shown that the specific sequences where LOC730101 binds to the BECN1 protein are 2001-3784nt of LOC730101 and 1-134aa of the BECN1 protein, and that LOC730101 inhibits the phosphorylation site of BECN1 by binding specifically to BECN1 and thus reducing BECN1 phosphorylation.

### LOC730101 promotes drug sensitivity in ovarian cancer through inhibition of autophagy

**Fig. 6:**
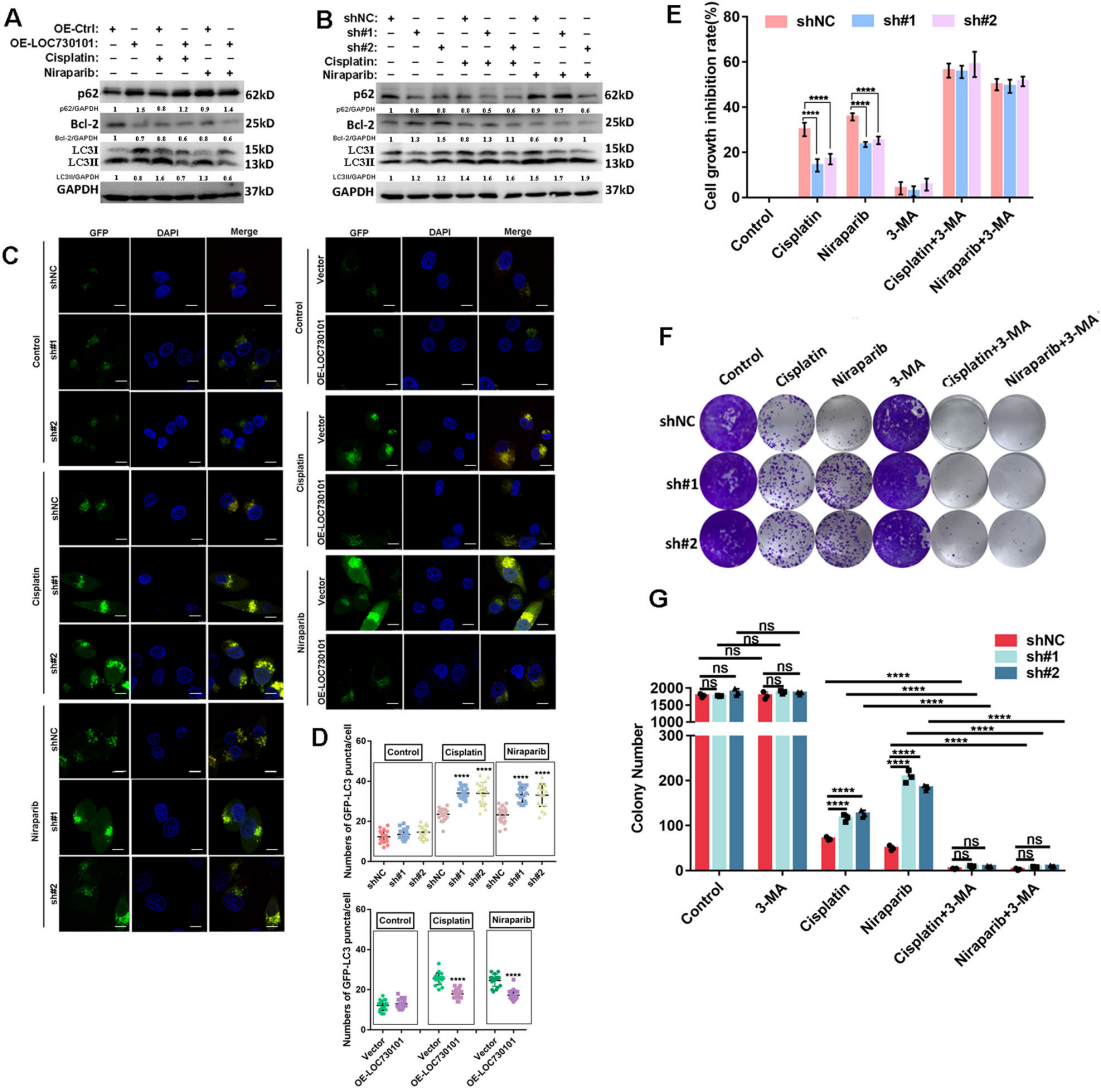
LOC730101 promotes drug sensitivity in ovarian cancer through inhibition of autophagy. **A** Ovarian cancer cells were treated with 10 µM cisplatin or 10 µM niraparib for 24 h, Western Blot was used to detect the expression of p62, Bcl2 and LC3 in control and overexpression LOC730101 OC cells, and GAPDH was used as an internal reference. **B** Western blot was used to detect the expression of p62, Bcl2 and LC3 in control and knockdown LOC730101 OC cells. GAPDH was used as an internal reference. **C** The GFP-LC3 dual fluorescent autophagy indicator system labels and tracks changes in LC3 as well as autophagic flow. Scale, 10 µm. **D** The statistical analysis of the numbers of GFP-LC3 puncta in OC cells, t-test, ****, *p* < 0.0001. **E** The shNC and shLOC730101 #1/#2 ovarian cancer cells were treated with 5 µM cisplatin, 5 µM niraparib, 0.25 mM 3-MA, 5 µM cisplatin and 0.25 mM 3-MA, 5 µM niraparib and 0.25 mM 3-MA respectively for 72 h, and the cell activity was detected by CCK8. t-test, **, *p* < 0.01, ****, *p* < 0.0001. **F** The shNC and shLOC730101 #1/#2 ovarian cancer cells were treated with 5 µM cisplatin, 5 µM niraparib, 0.25 mM 3-MA, 5 µM cisplatin and 0.25 mM 3-MA, 5 µM niraparib and 0.25 mM 3-MA respectively for 72 h, then replace the normal medium and continue to culture for 10 days. The cell proliferation was detected by colony-forming experiment. (**G**) The statistical of the numbers of cell colony cells. two-way ANOVA, ****, *p* < 0.0001.

The mechanism by which LOC730101 inhibits the formation of the autophagy complex BECN1-VPS34 by specifically binding to BECN1 has been clearly established, but what role does autophagy play in the treatment of ovarian cancer cells with drugs? The expression of autophagy degradation substrate p62, autophagy marker LC3 and anti-apoptotic protein Bcl2 were detected by Western Blot in stable overexpression and stable knockdown LOC730101 ovarian cancer cells treated with cisplatin and niraparib respectively. The results showed that after treatment with cisplatin and niraparib respectively, the anti-apoptotic protein Bcl2 was reduced, the autophagy substrate p62 was increased and the autophagy marker LC3 II was reduced in the high expression LOC730101 group compared to the control group, while the trend was reversed in the knockdown group (Fig. 6A, B). Immunofluorescence experiments combined with confocal microscopy also showed the same results (Fig. 6C, D). This indicates that autophagy was elevated in ovarian cancer cells after treatment with drugs, and high expression of LOC730101 could play a role in promoting drug sensitivity in ovarian cancer cells by inhibiting autophagy and promoting apoptosis. Autophagy plays a protective role in the process of drug treatment of ovarian cancer cells.

The autophagy inhibitor 3-methyladenine (3-MA) is a selective inhibitor of phosphatidylinositol 3 phosphokinase (PI3K) that acts on VPS34 and PI3Kγ, permanently inhibits type I PI3K but transiently inhibits type III PI3K and also exerts a blocking effect on autophagosome formation [33]. 3-MA has been widely used as an autophagy inhibitor in the literature [34]. We chose to use the autophagy inhibitor 3-MA to further demonstrate that LOC730101 exerts a role in promoting drug sensitivity in ovarian cancer cells by inhibiting autophagy and promoting apoptosis. When ovarian cancer cells were treated with cisplatin, niraparib, 3-MA, cisplatin and 3-MA, and niraparib and 3-MA, respectively, CCK8 assayed cellular activity. The proliferation inhibition rate of ovarian cancer cells treated with the combination of cisplatin and 3-MA or niraparib and 3-MA, respectively, was significantly higher than that of the group treated with cisplatin or niraparib alone (Fig. S5B). When ovarian cancer cells were treated with cisplatin or niraparib alone, respectively, the proliferation inhibition rate of ovarian cancer cells with knockdown of LOC730101 was lower than that of the control group; whereas when ovarian cancer cells were treated with the autophagy inhibitor 3-MA alone, the proliferation inhibition rate of ovarian cancer cells with knockdown of LOC730101 did not change compared to the control group, therefore 3-MA did not significantly inhibit cell proliferation. Whereas the proliferation inhibition rate of ovarian cancer cells with cisplatin and 3-MA or niraparib and 3-MA combination treatment of ovarian cancer cells, the proliferation inhibition rate of ovarian cancer cells was significantly higher than that of the group treated with cisplatin or niraparib alone, and the difference in proliferation inhibition rate reduced by knockdown of LOC730101 was reverted (Fig. 6E). This suggests that LOC730101 exerts its role in promoting drug sensitivity in ovarian cancer cells through inhibition of autophagy.

Similarly, when ovarian cancer cells were treated with cisplatin, niraparib, 3-MA, cisplatin and 3-MA, and niraparib and 3-MA, respectively, the colony-forming unit assays examined their survival and proliferation, and the results showed the same trend (Fig. 6F, G). Whereas the combination of treatment with 3-MA, the number of colonies formed in ovarian cancer cells treated with 3-MA was significantly lower than that in the group treated with cisplatin or niraparib alone, and the difference in colony formation increased by knockdown of LOC730101 was reverted (Figs. S5C and S5D). Apoptosis rates were detected by flow cytometry when control and overexpressed LOC730101 ovarian cancer cells were treated with cisplatin, niraparib, 3-MA, cisplatin and 3-MA, niraparib and 3-MA, respectively. The results from the apoptosis rates showed that when the combination of cisplatin and 3-MA or niraparib and 3-MA treatment resulted in significantly more apoptosis of ovarian cancer cells than the group treated with cisplatin or niraparib alone, and the difference in apoptosis rate reduced by knockdown of LOC730101 was reverted (Fig. S5A, S5E). These results suggest that LOC730101 promotes drug sensitivity in ovarian cancer cells by inhibiting autophagy and that autophagy inhibitors combined promotes significantly drug sensitivity of ovarian cancer cells to cisplatin or niraparib.

### LOC730101 inhibits DNA damage repair by affecting autophagy-regulated histone H2A ubiquitination

**Fig. 7:**
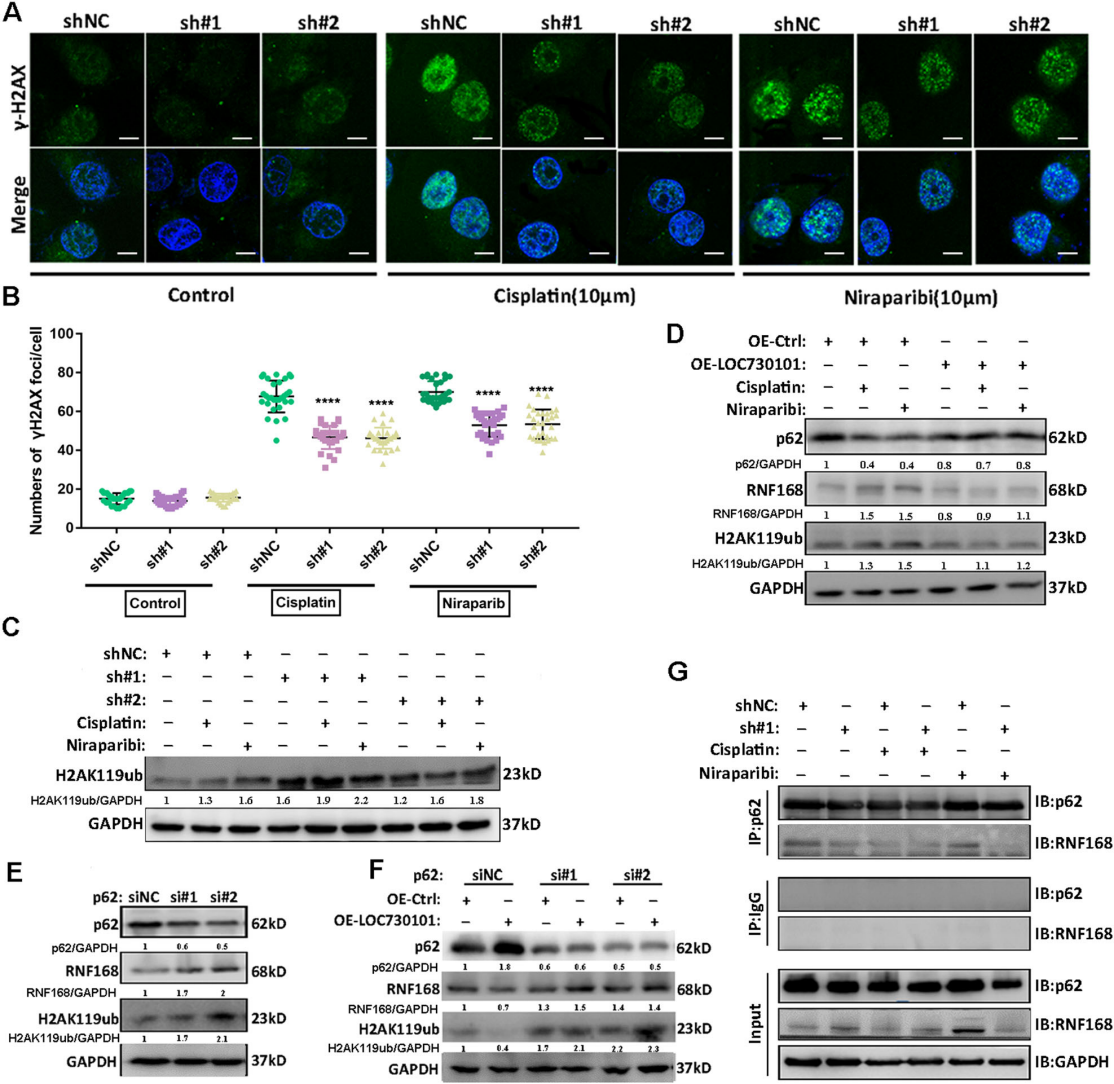
LOC730101 inhibits DNA damage repair by inhibiting autophagy and regulating histone H2A ubiquitination. **A** After the shNC and shLOC730101 #1/#2 ovarian cancer cells were treated with 10 µM cisplatin or 10 µM niraparib respectively, immunofluorescence was used to detect the expression of DNA damage markers γ-H2AX (green), DAPI labeled nucleus (blue). Scale, 10 µm. **B** The statistical of the numbers of γ-H2AX foci in OC cells, t-test, ****, p < 0.0001. **C** After the shNC and shLOC730101 #1/#2 ovarian cancer cells were treated with 10 µM cisplatin or 10 µM niraparib respectively for 24 h, the expression of H2AK119ub was detected by Western Blot, and GAPDH was used as the internal reference. **D** After treating the control ovarian cancer cells Ctrl and OE-LOC730101 with 10 µM cisplatin or 10 µM niraparib respectively for 24 h, Western Blot was used to detect the protein expression of p62, RNF168 and H2AK119ub, and GAPDH was used as the internal reference. **E** After p62 was knocked down instantaneously, the expression of p62, RNF168 and H2AK119ub was detected by Western Blot, and GAPDH was the internal reference. **F** After p62 was knocked down instantaneously, Western Blot was used to detect the expression of p62, RNF168 and H2AK119ub in Ctrl and OE-LOC730101 ovarian cancer cells, GAPDH was used as an internal reference. **G** After the control and knockdown LOC730101 ovarian cancer cells were treated with 10 µM cisplatin or 10 µM niraparib respectively for 24 h, the expression of p62 and RNF168 in the complexes precipitated by Input, IgG antibody and p62 antibody was detected by Co-IP experiment combined with Western Blot.

Because cisplatin and niraparib have different mechanisms of cell-killing action, but both ultimately lead to DNA damage. Therefore, we hypothesized that LOC730101 might exert its effect to promote drug sensitivity in ovarian cancer cells by affecting DNA damage. To further identify the mechanism of action by which LOC730101 promotes increased drug sensitivity in ovarian cancer cells, immunofluorescence assays combined with confocal observation revealed that after treatment of ovarian cancer cells with cisplatin or niraparib drugs, respectively, the DNA damage marker γ-H2AX focus was reduced in ovarian cancer cells with LOC730101 knock down compared to the control group (Fig. 7A, B). It was suggested that LOC730101 could promote DNA damage in ovarian cancer cells after drug treatment.

It has been documented that autophagy can affect DNA damage repair caused by radiotherapy [20]. P62 is a substrate and receptor for ubiquitin and LC3 proteins as selective autophagy, degrading ubiquitinated substrates through autophagy. When cellular autophagy is insufficient, it leads to p62 accumulation, which inhibits the ubiquitination of histone H2A by the ubiquitin ligase RNF168, ultimately affecting the recruitment of DNA damage repair factors such as RAD51 and BRCA1 at the damage site and preventing DNA repair [20]. Autophagy also ensures proper regulation of the DNA repair pathway by RNF168 through degradation of the deubiquitinating enzyme USP14 [19]. To demonstrate the mechanism of association between LOC730101, autophagy and DNA damage, we examined the regulatory relationship between LOC730101 and histone H2A ubiquitination before and after drug treatment by Western Blot and showed that H2AK119ub increased in ovarian cancer cells after treatment with cisplatin or niraparib, respectively, while knockdown LOC730101 was significantly increased in ovarian cancer cells with H2AK119ub compared to control (Fig. 7C), suggesting that LOC730101 can be involved in DNA damage repair through inhibition of histone H2A ubiquitination.

It has been reported that the autophagic substrate p62 can inhibit the ubiquitination of histone H2A by the ubiquitin ligase RNF168. We then examined the relationship between LOC730101, autophagy and histone H2A ubiquitination before and after drug treatment by Western Blot. The results showed that the overexpression of LOC730101 significantly increased the expression of p62, and decreased the expression of RNF168 and H2AK119ub compared with the control group after the treatment of ovarian cancer cells with cisplatin and niraparib respectively (Fig. 7D). This suggests that LOC730101 may be involved in DNA damage repair by inhibiting autophagy and thus histone H2A ubiquitination. Based on the reports, we further explored the specific mechanism of association between the autophagic substrate p62 and the ubiquitin ligase RNF168. Western Blot assay revealed that knockdown of p62 increased RNF168 expression (Fig. 7E), indicating that p62 negatively regulates RNF168. Western Blot assay showed that p62 was increased and RNF168 was decreased in ovarian cancer cells endogenously overexpressing LOC730101 compared to the control. When p62 was transiently knocked down, p62 was decreased in both control and ovarian cancer cells overexpressing LOC730101, while the decreased RNF168 was reverted in ovarian cancer cells overexpressing LOC730101 (Fig. 7F). The Co-IP assay further confirmed that stable knockdown of LOC730101 inhibited the binding of p62 to RNF168 (Fig. 7G), suggesting that LOC730101 affects ubiquitin ligase RNF168 and thus histone H2A ubiquitination through p62. Above results suggest that LOC730101 plays a role in promoting drug sensitivity in ovarian cancer cells by inhibiting DNA damage repair involving histone H2A ubiquitination through the inhibition of autophagy.

### LOC730101 promotes drug sensitivity in ovarian cancer through inhibition of autophagy in *vivo*

**Fig. 8:**
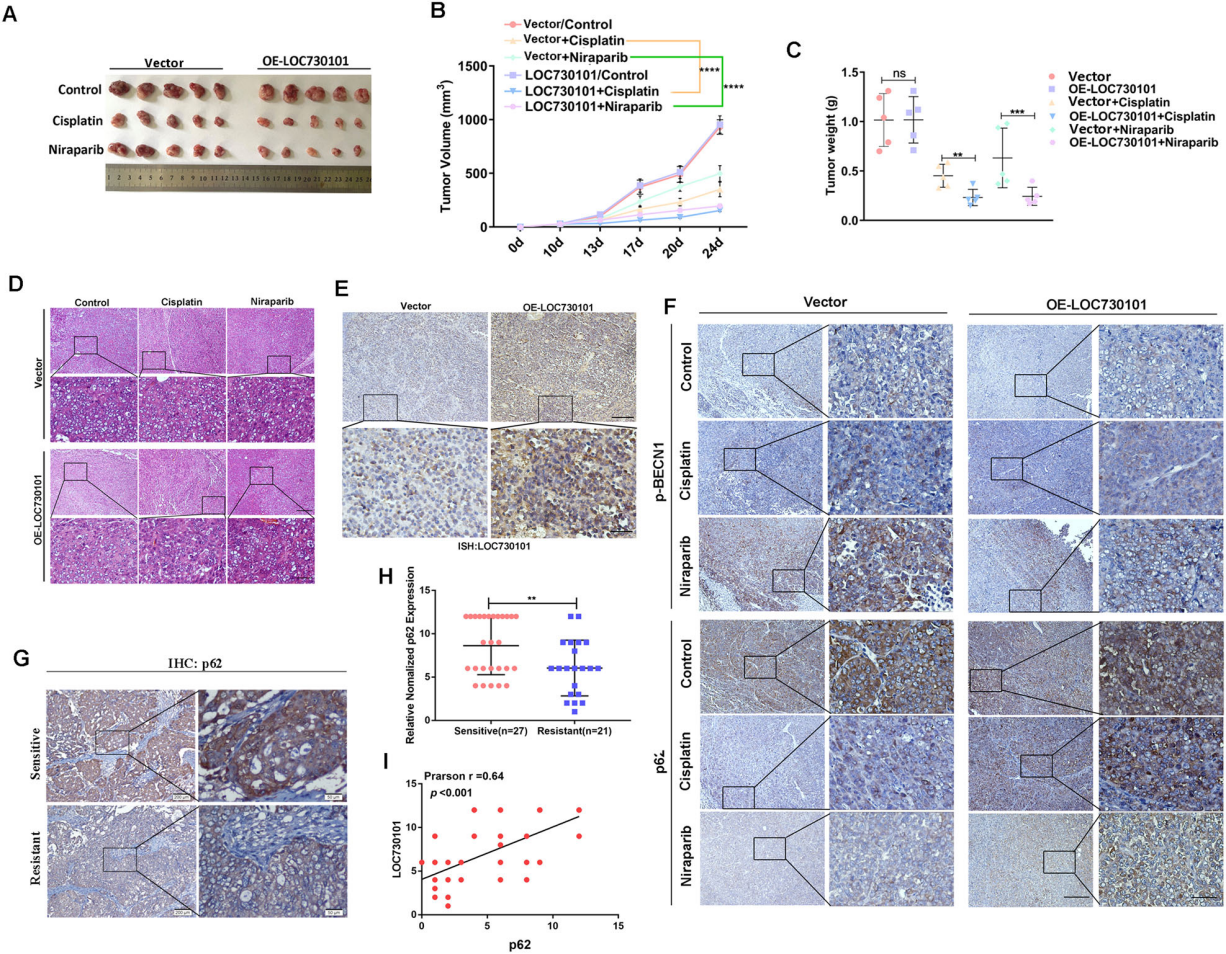
LOC730101 promotes drug sensitivity in ovarian cancer through inhibition of autophagy in vivo. **A** Photographs of stripped subcutaneous tumors in tumor-bearing nude mice in the OVCAR3 Vector and OVCAR3 OE-LOC730101 groups treated with saline, cisplatin and niraparib, respectively. **B** Growth curves of subcutaneous tumors in nude mice. t-test, ****, *p* < 0.0001. **C** Subcutaneous weight statistics of subcutaneous tumors in nude mice. t-test, **, *p* < 0.01, ***, *p* < 0.001. **D** H&E staining to examine the morphological structure of cells in paraffin sections of nude mouse subcutaneous tumors. Scale bars, 100 µm and 20 µm. **E** In situ hybridization staining experiments were performed to detect LOC730101 expression in paraffin sections of subcutaneous tumors in the OVCAR3 Vector and OVCAR3 OE-LOC730101 groups, respectively. Scale bars, 100 µm and 20 µm. **F** Immunohistochemical assay to detect the expression of p-BECN1 and p62 in paraffin sections of subcutaneous tumors in nude mice. Scale bars, 100 µm and 20 µm. **G** Immunohistochemical assay to detect the expression of p62 in platinum-sensitive ovarian cancer tissues (n = 27) and platinum-resistant ovarian cancer tissues (n = 21). Scale bars, 100 µm and 20 µm. **H** Statistical plots of p62 in platinum-sensitive ovarian cancer tissues (n = 27) and platinum-resistant ovarian cancer tissues (n = 21) for scoring immunohistochemical assay. t-test, **, *p* < 0.01. **I** Correlation analysis of p62 and LOC730101 expression in platinum-sensitive ovarian cancer tissues (n = 27) and platinum-resistant ovarian cancer tissues (n = 21). Pearson r = 0.64, *p* < 0.01.

We validated the role and mechanism of LOC730101 in promoting drug sensitivity in ovarian cancer through inhibition of autophagy using a subcutaneous xenograft model of ovarian cancer in nude mice. We evaluated the response of ovarian cancer cells with overexpressing LOC730101 or without to cisplatin or niraparib drugs. It was shown that the growth of subcutaneous tumors in OE-LOC730101 group was significantly inhibited compared with that in Ctrl group, as well as the growth rate, size and weight of subcutaneous tumors in the OE-LOC730101 group were significantly smaller than those in the Vector group, indicating that the OE-LOC730101 group was more sensitive to cisplatin or niraparib than those in the control group (Fig. 8A–C).

H&E staining analysis revealed the morphological structure of subcutaneous tumors in six groups of nude mice (Fig. 8D). In situ hybridization experiments revealed significantly higher LOC730101 expression in subcutaneous tumors in the OE-LOC730101 group compared to the Vector group (Fig. 8E). Immunohistochemical results showed that the subcutaneous tumors in Vector group and OE-LOC730101 group, treated with cisplatin or niraparib, respectively, had increased expression of p-BECN1, RNF168, H2AK119ub and γ-H2AX, but compared with OVCAR3 Vector group, the expression of p-BECN1, RNF168, H2AK119ub and H2AX in OVCAR3 OE-LOC730101 group increased less; However, the expression of p62 in subcutaneous tumors of OVCAR3 Vector group and OVCAR3 OE-LOC730101 group treated with cisplatin or niraparib respectively decreased, but compared with OVCAR3 Vector group, the expression of p62 in OVCAR3 OE-LOC730101 group decreased less.(Fig. 8F, Fig. S6A and S6B). The mechanism of LOC730101 in promoting drug sensitivity in ovarian cancer through inhibition of autophagy inhibiting histone H2A ubiquitination-mediated DNA damage repair was further validated by subcutaneous tumor-forming in vivo experiments in nude mice.

### Clinical sample validates that LOC730101 promotes ovarian cancer drug sensitivity through inhibition of autophagy

We then analyzed the expression of p62 in 27 cases of platinum-sensitive OC tissues and 21 cases of platinum-resistant OC tissues. We found that p62 were highly expressed in platinum-sensitive OC compared with platinum-resistant OC tissues (Fig. 8G, H). And the correlation analysis of LOC730101 and p62 demonstrated that the expression of LOC730101 and p62 was significantly positively correlated (Pearson r = 0.64, P < 0.0001) (Fig. 8I). Finally, we downloaded the GSE51373 ovarian cancer data from the GEO database and found that p62 expression was up-regulated in ovarian cancer platinum-sensitive tissues compared to the platinum-resistant group (Fig. S6C). Moreover, analysis by the TCGA database revealed that high p62 expression was positively correlated with progression-free survival in ovarian cancer patients (Fig. S6D), suggesting that ovarian cancer patients with high p62 expression have a better prognosis, further suggesting that LOC730101 promotes ovarian cancer drug sensitivity through inhibition of autophagy.

## Discussion

**Fig. 9:**
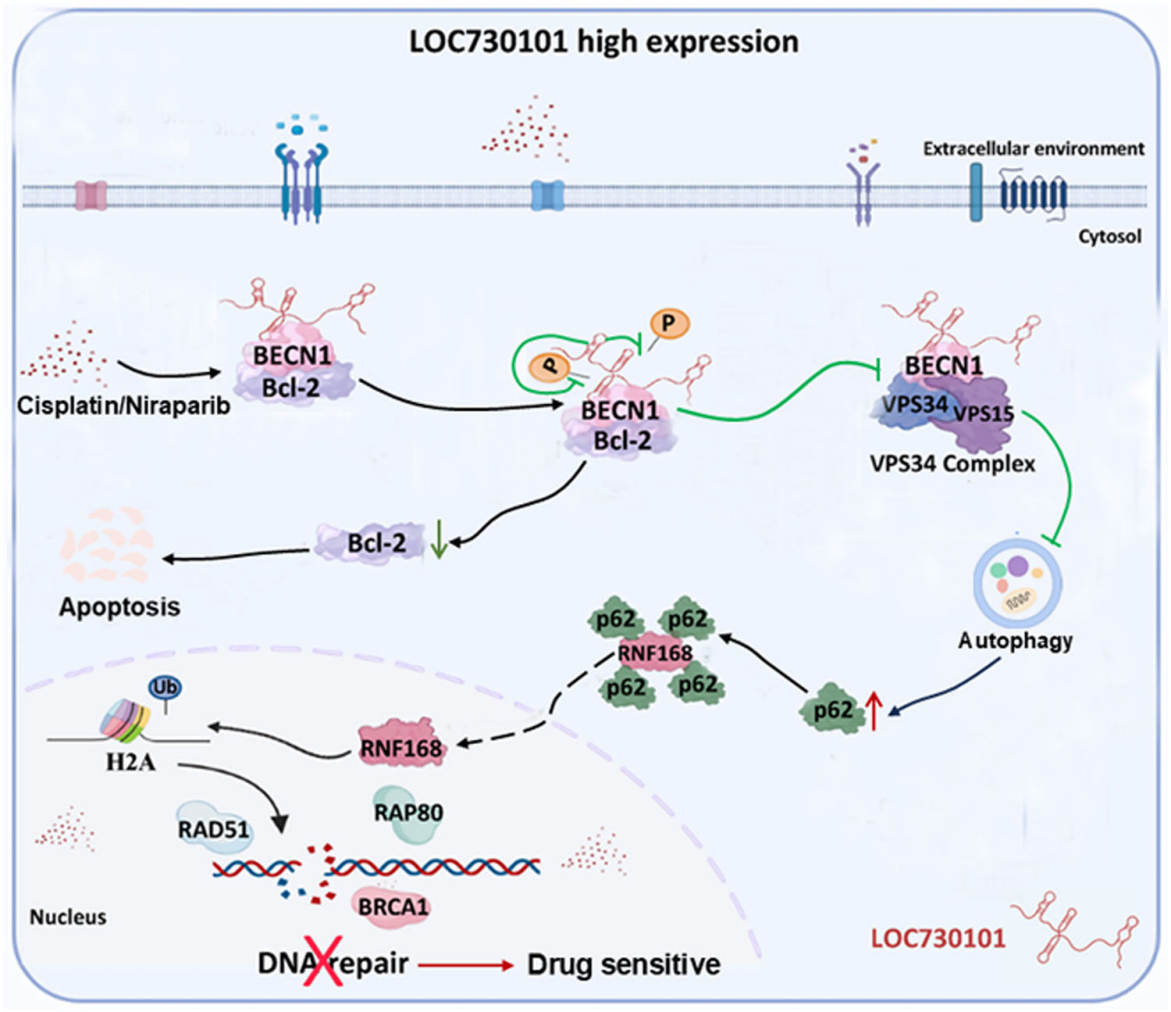
A working model for the role and mechanism of LOC730101 in promoting drug sensitivity of ovarian cancer. LOC730101 specifically binds to BECN1, a key initiator of autophagy in ovarian cancer cells. When cells were treated with cisplatin or niraparib, high expression of LOC730101 reduces BECN1 phosphorylation and inhibits the dissociation of BECN1-Bcl2 complex, leading to reduced autophagosome BECN1-VPS34 formation. Then, the accumulation of the autophagy substrate p62 is increased, which prevents RNF168 bound to p62 from being released into the nucleus, thus inhibiting the ubiquitination of histone H2A, and ultimately leading to the failure of recruiting DNA damage factors, such as BRCA1, RAD51 and RAP80, to play the role in DNA damage repair. As a result, DNA damage in ovarian cancer cells could not be repaired, and ovarian cancer cells eventually died from the killing effect of cisplatin or niraparib, and the sensitivity of ovarian cancer cells to drugs increased.

In our research, we found the role and mechanism of action of LOC730101 in promoting drug sensitivity in ovarian cancer. We found that LOC730101 specifically binds to the autophagy key protein BECN1 in ovarian cancer cells. When cells were treated with cisplatin or niraparib, high expression of LOC730101 reduces BECN1 phosphorylation and inhibits the dissociation of BECN1-Bcl2 complex, leading to reduced autophagosome BECN1-VPS34 formation. Then, the accumulation of the autophagy substrate p62 is increased, which prevents RNF168 bound to p62 from being released into the nucleus, thus inhibiting the ubiquitination of histone H2A, and ultimately leading to the failure of recruiting DNA damage factors, such as BRCA1, RAD51 and RAP80, to play the role in DNA damage repair. As a result, DNA damage in ovarian cancer cells could not be repaired, and ovarian cancer cells eventually died from the killing effect of cisplatin or niraparib, and the sensitivity of ovarian cancer cells to drugs increased. (Fig. 9).

LncRNAs can act through a number of different mechanisms such as chromatin remodeling, chromatin interactions, ceRNA and natural antisense transcripts in cancer [35]. The lncRNA in the nucleus can control the epigenetic state of genes, participate in transcriptional regulation, and also involve variable splicing processes, while the lncRNA in the cytoplasm is mainly involved in post transcriptional regulation, including influencing the stability of mRNA, translation regulation, and ceRNA [36]. There are only two reports in the literature on LOC730101, one of which found that LOC730101 promotes cell cycle progression and ultimately cell proliferation and growth by enhancing the activity of the Wnt/β-catenin signaling pathway [37]. Another one indicated that LOC730101 expression was closely associated with the carcinogenesis of osteosarcoma [38]. Our previous analysis of ovarian cancer databases revealed that LOC730101 expression tended to be down-regulated with increasing malignancy of ovarian cancer, was highly expressed in platinum-sensitive tissues of ovarian cancer and was positively correlated with prognosis, and that LOC730101 may function as an oncogenic function in ovarian cancer, but studies on the mechanism of action of LOC730101 are superficial and have not been reported in ovarian cancer. In this paper, we found that LOC730101 was distributed in the nucleoplasm, and based on the results of RNA pull-down experiments and mass spectrometry identification, we finally found that the autophagy key protein BECN1 is the downstream target of LOC730101.

LOC730101 regulates BECN1 through a way of post-translational modifications (PTMs). PTMs of BECN1 are critical for autophagy control and regulation. In addition to DAPK, which phosphorylates BECN1 on Thr119 of the BH3 structural domain, it has also been reported that AMPK can phosphorylate BECN1 at S90 and S93, which also contributes to the activation of the VPS34 complex [39]. This further explains the finding in this paper that high expression of LOC730101 can inhibit the activation of the VPS34 complex by suppressing p-BECN1^Ser93^ and ultimately inhibit the onset of autophagy. In combination with our findings, we show that LOC730101 acts in a manner that regulates post-translational modifications by inhibiting phosphorylation of BECN1. Thr119 and Ser93 phosphorylation of BECN1 may be used as a predictive marker for poor prognosis in ovarian cancer, as well as a marker for the potential benefit of ovarian cancer drug therapy and early identification of drug resistance.

Numerous studies have shown that the development of drug resistance in ovarian cancer is related to the activation of autophagy. Chemotherapy can usually induce a stress response in cancer cells, leading to increased expression of autophagy-related genes or proteins in cancer cells, which eventually manifests itself in the form of reduced sensitivity of tumor cells to chemotherapeutic agents and the emergence of drug resistance. Cisplatin is the first-line therapeutic agent used for the treatment of ovarian cancer; however, cisplatin resistance is a major cause of treatment failure in ovarian cancer patients [40]. Autophagy can lead to cisplatin resistance, and it has been noted that NF-E2-related factor 2 (Nrf2) can lead to cisplatin resistance through activation of autophagy in ovarian cancer [40]. In addition, elevated expression of the oncogenic promoter methionine synthase reductase (MTRR) has been found in ovarian cancer, and cisplatin resistance can be inhibited by inhibiting MTRR expression which can reduce autophagy [41]. The oncoprotein YAP induces cisplatin resistance through activation of autophagy in human ovarian cancer cells [42]. These results all suggest that autophagy plays a protective role in cisplatin treatment of ovarian cancer. Phosphatase 2 A catalytic subunit (PP2Ac), a target of cisplatin, inhibits the accumulation of LC3 II and restores p62, and knockdown of PP2Ac promotes autophagy in cisplatin-resistant ovarian cancer cells, suggesting that protective autophagy inhibited by PP2Ac is also a partial mechanism of cisplatin resistance in ovarian cancer [43]. In addition, cisplatin activates ERK and promotes ERK-induced autophagy, thereby counteracting cisplatin-induced cell death, and knockdown of ERK reduces cisplatin-induced autophagy and increases cisplatin-induced cell death [44]. Paclitaxel has also been identified as a first-line chemotherapeutic agent against ovarian cancer, but chemoresistance still exists [45]. Studies have shown that up-regulation of autophagy acts as a promoter of paclitaxel resistance in ovarian cancer induced by the high expression of the autophagy-associated gene TXNDC17 [16]. Autophagy has a protective role in regulating cellular chemo-sensitivity to cisplatin, specially in human ovarian cancer and can be inhibited by autophagy inhibitors and BECN1 small interfering RNA [46, 47]. In this paper, our study also illustrates that combined autophagy inhibitors promote the sensitivity of ovarian cancer cells to cisplatin and niraparib, and that high expression of LOC730101 exerts an inhibitory effect on autophagy, which further elucidates the mechanism of action of LOC730101 in promoting drug sensitivity in ovarian cancer cells through the inhibition of protective autophagy.

At present, chloroquine (CQ) and hydroxychloroquine (HCQ) are listed in the list of essential drugs of the World Health Organization as safe and effective drugs required by the health system [48]. CQ destroys/interrupts autophagy-lysosomal fusion at the initial stage [49] and enhances the anti-proliferation effect of chemotherapy drugs [50, 51]. A small trial involving 18 patients with glioblastoma provided clinical evidence of improved prognosis, with significantly longer median survival in patients treated with CQ through autophagy inhibition in combination with radiation compared with controls (33 months compared with 11 months) [52].Other early studies combining CQ with radiotherapy for brain metastases have also shown improved intracranial tumor control [52]. There are also several early clinical trials on HCQ in patients with different malignancies (e.g., melanoma, breast cancer, non-small cell lung cancer, etc.) involving multiple drug combinations [53]. With further studies on the clinical use of autophagy inhibitors, autophagy inhibitors in combination with chemotherapy or in combination with targeted agents are potentially effective treatment strategies for patients with drug-resistant ovarian cancer in the future. Increased autophagy in ovarian cancer cells can promote the development of their drug resistance; therefore, blocking the production of autophagy or reducing the effect of autophagy may be an important therapeutic option to improve the sensitivity to chemotherapy and increase the cytotoxicity of chemotherapeutic agents in ovarian cancer patients, especially those with drug-resistant ovarian cancer.

In this paper, we analyzed the GEO database and TCGA database and found that LOC730101 was highly expressed in platinum-sensitive tissues of ovarian cancer and that patients with high expression had better progression-free survival. A trend of low expression of LOC730101 was also detected in drug-resistant ovarian cancer cells, and ex vivo experiments also demonstrated that high expression of LOC730101 promoted ovarian cancer sensitivity to cisplatin and PARP inhibitors. This suggests that LOC730101 may be used as a prognostic marker to predict the sensitivity of ovarian cancer to platinum and PARP inhibitors.

## Supplementary information

Supplementary materials

WB raw data

## Data Availability

The data supporting the findings of this study are available from the corresponding author upon reasonable request.
