# Supplementary material for: LOC730101 improves ovarian cancer drug sensitivity by inhibiting autophagy-mediated DNA damage repair via BECN1
Source: Cell Death Dis. 2024 Dec 18;15(12):893. doi: 10.1038/s41419-024-07278-1 (PMC11655529; doi:10.1038/s41419-024-07278-1)
Supplement: Supplementary file 1 — Supplementary materials [file 41419_2024_7278_MOESM1_ESM.pdf]

LOC730101 improves ovarian cancer drug sensitivity by inhibiting autophagy-mediated DNA damage repair via BECN1

Yancheng Zhong[1,2,3#], Yang Shuai[4#], Juan Yang[5], Mojian Zhang[1,3], Tiantian He[1,3], Leliang Zheng[1,3], Sheng Yang[6*], Shuping Peng[1,3*]

Supplementary materials

Figure S1

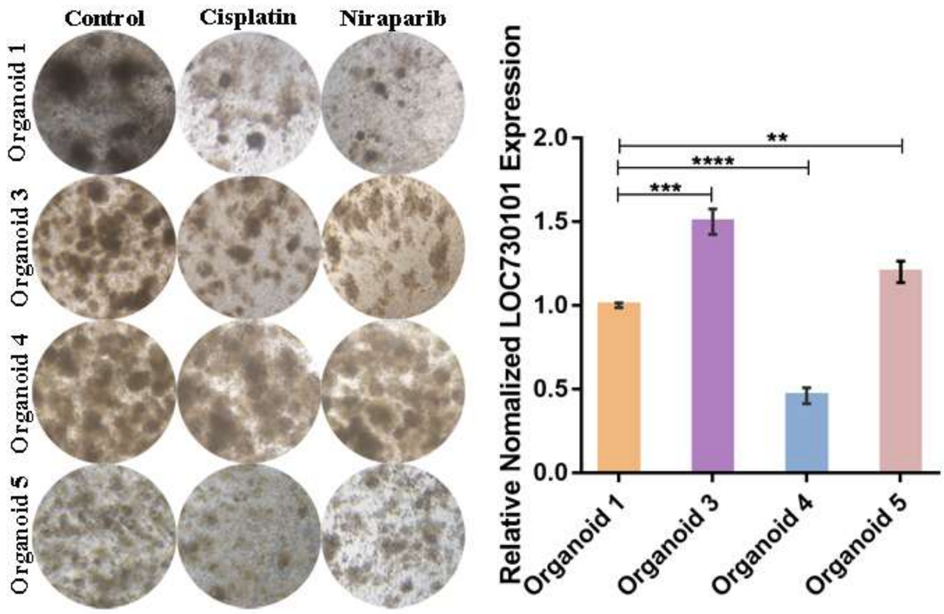

Figure S1. Morphogram of an ovarian cancer-derived organoid after cisplatin and niraparib treatment. T-test, **, $p < 0.01$, ***, $p < 0.001$, ****, $p < 0.0001$.

Figure S2

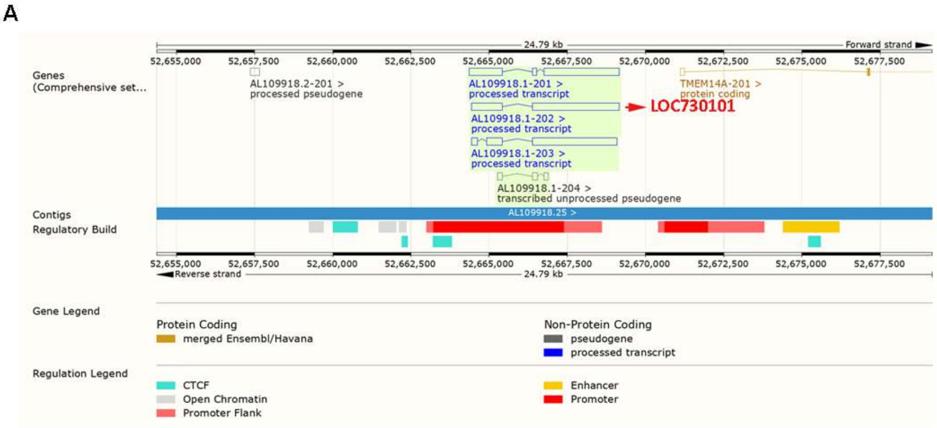

B

Coding Potential Assessment Tool

Result for species name : hg19 with job ID :1593488166							
Data ID	Sequence Name	RNA Size	ORF Size	Ficket Score	Hexamer Score	Coding Probability	Coding Label
0	NR_024403.1	3784	285	0.5087	-0.160519406042	0.0089524639757116	no

Figure S2. Basic information about LncRNA LOC730101. (**A**) LOC730101's location in the genome and transcript information. (**B**) Online CAPT website predicts the coding capacity of LOC730101.

Figure S3

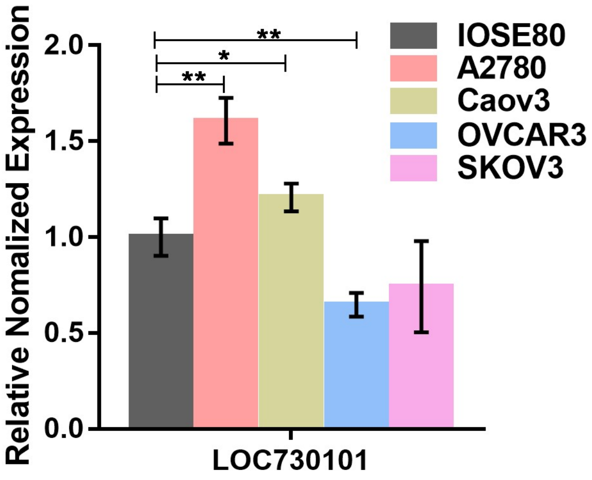

Figure S3. Expression of LOC730101 was detected by qPCR in ovarian normal epithelial cells IOSE80 and ovarian cancer cells A2780, Caov3, OVCAR3 and SKOV3. t-test, *, $p < 0.05$, **, $p < 0.01$.

Figure S4

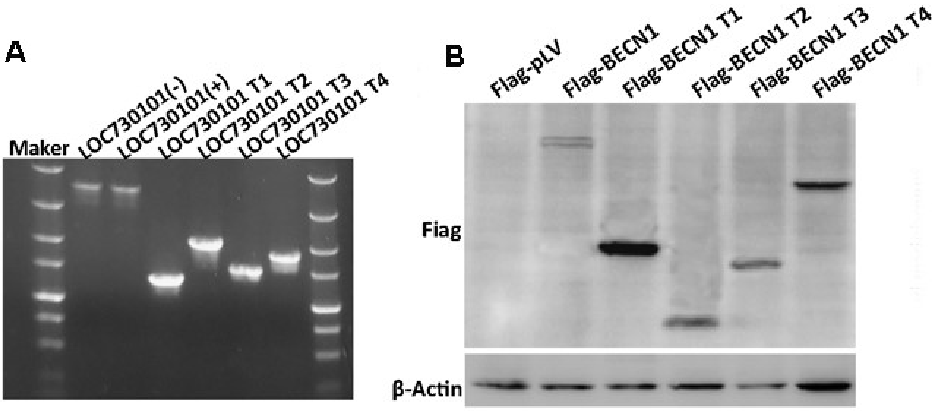

Figure S4. (**A**) Results of gel electrophoresis of DNA from LOC730101 positive and antisense strands and their deletion mutant plasmids. (**B**) Western Blot detection of protein expression of Flag-BECN1 full-length and deletion mutants Flag-BECN1 T1, Flag-BECN1 T2, Flag-BECN1 T3 and Flag-BECN1 T4, with β-actin as an internal reference.

Figure S5

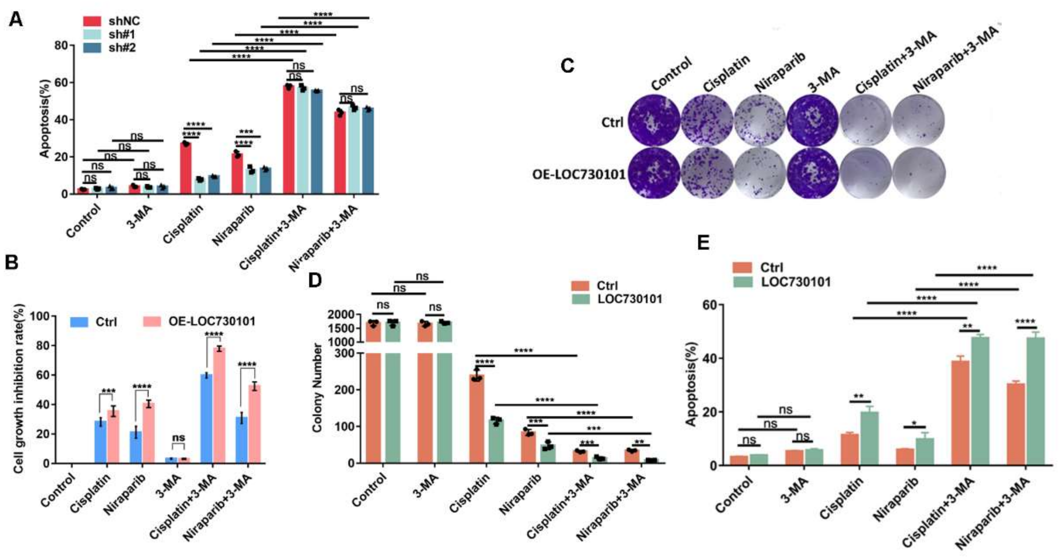

Figure S5. (**A**) The shNC and shLOC730101 #1/#2 ovarian cancer cells were treated with 10 μM cisplatin, 10 μM niraparib, 1 mM 3-MA, 10 μM cisplatin and 1 mM 3-MA, 10 μM niraparib and 1 mM 3-MA respectively for 48 h, then flow cytometry was used to detect the apoptosis rate. two-way ANOVA, ***, p < 0.001, ****, p < 0.0001. (**B**) Cell activity of Ctrl and OE-LOC730101 ovarian cancer cells was detected by CCK8. two-way ANOVA, ***, p < 0.001, ****, p < 0.0001. (**C**) Clone formation assay to detect cell proliferation of Ctrl and OE-LOC730101 ovarian cancer cells. (**D**) Statistical plots of the number of clones formed. (**E**) Statistical plots of apoptosis rates detected by flow cytometry. two-way ANOVA, *, p < 0.05, **, p < 0.01, ***, p< 0.001, ****, p < 0.0001.

Figure S6

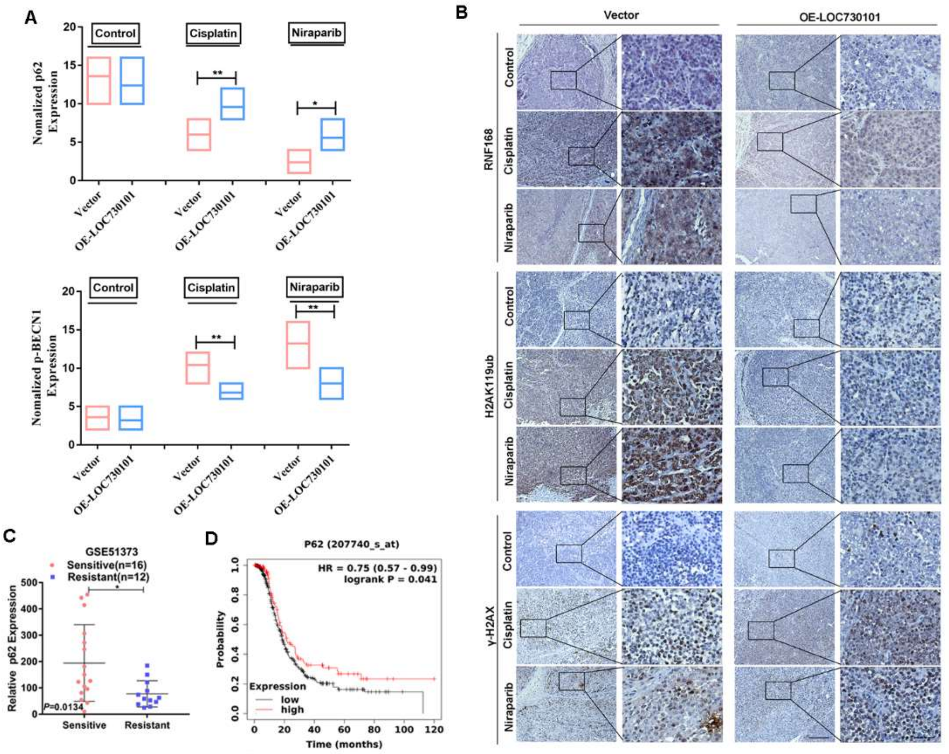

Figure S6. (**A**) The statistics of immunohistochemical results, t-test, *, $p < 0.05$, **, $p < 0.01$. (**B**) Immunohistochemical assay to detect the expression of RNF168, H2AK119ub and γ-H2AX in paraffin sections of nude mice with subcutaneous tumors. Scale bars, 100 μm and 20 μm. (**C**) GSE51373 data analysis results showed that the expression of p62 in platinum sensitive tissues (n=16) of ovarian cancer was higher than that in platinum resistant tissues (n=12), p=0.0134. (**D**) The survival curve showed that the expression of p62 was positively correlated with the progression free survival of ovarian cancer patients, p=0.041.

Table S1

Table S1. Constructs sequences

Name	Sequence（5'-3'）
LOC730101(-)-F	TTTAACTCGAGATAAAGCGCGCCCG
	CTGCTCCCACAAAGCCGGGCTGT
LOC730101(-)-R	CCGAGAATTCGCAAAGGAAGCCAATTTTATTGAAATGCA

LOC730101(+)-F	TTTAAGAATTCATAAAGCGCGCCCGCTGCTCCCA<br>CAAAGCCGGGCTGT
LOC730101(+)-R	CCGACTCGAGGCAAAGGAAGCCAATTTTATTGAAATGCA
LOC730101 T1-R	CCGACTCGAGCAAAGGCAAGAAAGGAGGTGAG
LOC730101 T2-R	CCGACTCGAGTTAGAAAACATGGTCACAATA
LOC730101 T3-F	TTTAAGAATTCGAGGGCCGCCTTTTCTGAGTT
LOC730101 T3-R	CCGACTCGAGGAAGTTTGTAGACTGAATGC
LOC730101 T4-F	TTTAAGAATTCGAGGTCAGATAATGAGAATGG
BECN1-F	CCGGAATTCATGGATTACAAGGACGA<br>CGATGACAAGGAAGGGTCTAAGACGTCCAAC
BECN1-R	ATAAGAATGCGGCCGCTCATTTGTTATAAAATTGTGAGG
BECN1-T1-R	ATAAGAATGCGGCCGCTCAGCCGCCATCAGATGCCTCC
BECN1-T2-R	ATAAGAATGCGGCCGCTCAGTGATCCACATCTGTCTGG
BECN1-T3-F	CCGGAATTCATGGATTACAAG<br>GACGACGAGCCACTCTGTGAGGAATGCA
BECN1-T3-R	ATAAGAATGCGGCCGCTCACAGCTGCGTCTGGGCATAA

Table S2

Table S2. Sequences of siRNAs and shRNAs

Name	**Sequence（5'-3'）**
Scrambled siRNA	TTCTCCGAACGTCACGTTT
siLOC730101#1	ACAGGTGAATGCTTCTAAACC
siLOC730101#2	AGTGAAAGCTTTGGGAAATCC
siLOC730101#3	GTGTTAGAAGCAAGTTAGAGG
siP62#1	GCATTGAAGTTGATATCGA
siP62#2	CATCCAGTATTCAAAGCAT
siP62#3	ACAGATGGAGTCGGATAAC
shLOC730101#1-F	CCGGTACAGGTGAATGCTTCTAAAC<br>CTTCAAGAGAGGTTTAGAAGCATTCACCTGT TTTTTG
shLOC730101#1-R	AATTCAAAAAACAGGTGAATGCTTCT<br>AAACCTCTCTTGAAGGTTTAGAAGCATTCACCTGTA

shLOC730101#2-F	CCGGTAGTGAAAGCTTTGGGAAATCC
	TTCAAGAGAGGATTTCCCAAAGCTTTCACTTTTTTG
shLOC730101#2-R	AATTCAAAAAAGTGAAAGCTTTGGGAA
	ATCCTCTCTTGAAGGATTTCCCAAAGCTTTCACTA

Table S3

Table S3. Primers for qPCR

**Primmer Name**	**Primer sequence（5'-3'）**
LOC730101-F	TCTCACCCTACCGTTCGTCT
LOC730101-R	AGTGGCTTTGGGAGTTCAGG
BECN1-F	CTCCCGAGGTGAAGAGCATC
BECN1-R	GCTGTTGGCACTTTCTGTGG
GAPDH-F	AACGGATTTGGTCGTATTGG
GAPDH-R	TTGATTTTGGAGGGATCTCG
Actin-F	CTGGGACGACATGGAGAAAA
Actin-R	AAGGAAGGCTGGAAGAGTGC

Table S4

Table S4. Digoxin tag probe sequences for LOC730101

**Name**	**Sequence（5'-3'）**
LOC730101 probe#1	CTTTGGTGAGTTTGGGGAGGTGAATTTCTCCCCAGGATAT
LOC730101 probe#2	GTAGTTCAGAATGGTCATCCATCCATCGTTGCATCATTCA
LOC730101 probe#3	CCATCTATGTACAACTTACGAAAACTTGCATTATTATAGA
