# Supplementary material for: LOC730101 improves ovarian cancer drug sensitivity by inhibiting autophagy-mediated DNA damage repair via BECN1
Source: Cell Death Dis. 2024 Dec 18;15(12):893. doi: 10.1038/s41419-024-07278-1 (PMC11655529; doi:10.1038/s41419-024-07278-1)
Supplement: Supplementary file 2 — WB raw data [file 41419_2024_7278_MOESM2_ESM.pdf]

Fig. 4E

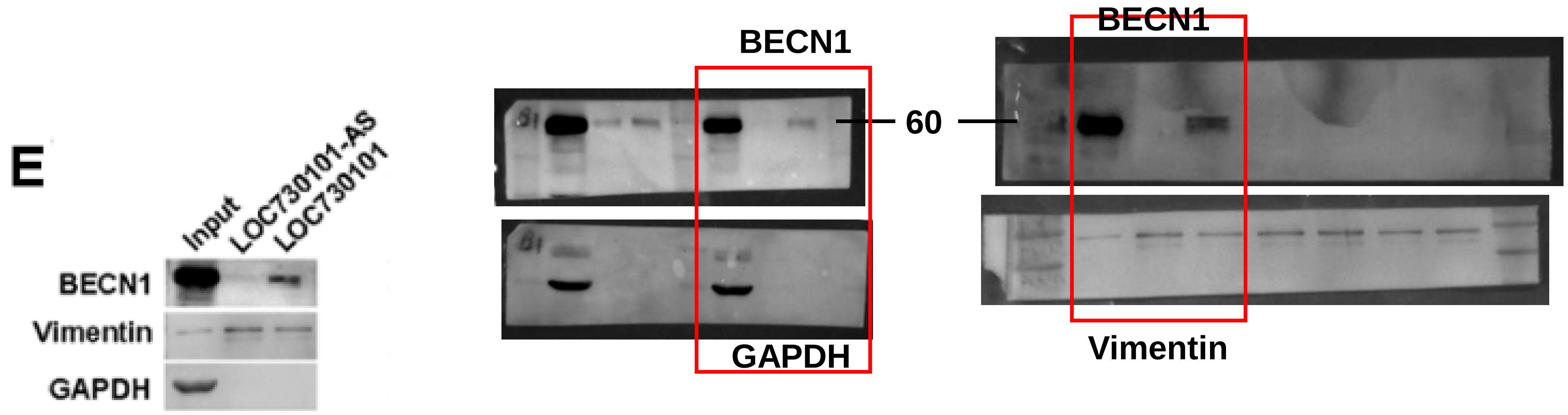
E
Input
LOC730101-AS
LOC730101
BECN1
Vimentin
GAPDH
BECN1
60
GAPDH
BECN1
Vimentin

Fig. 4H

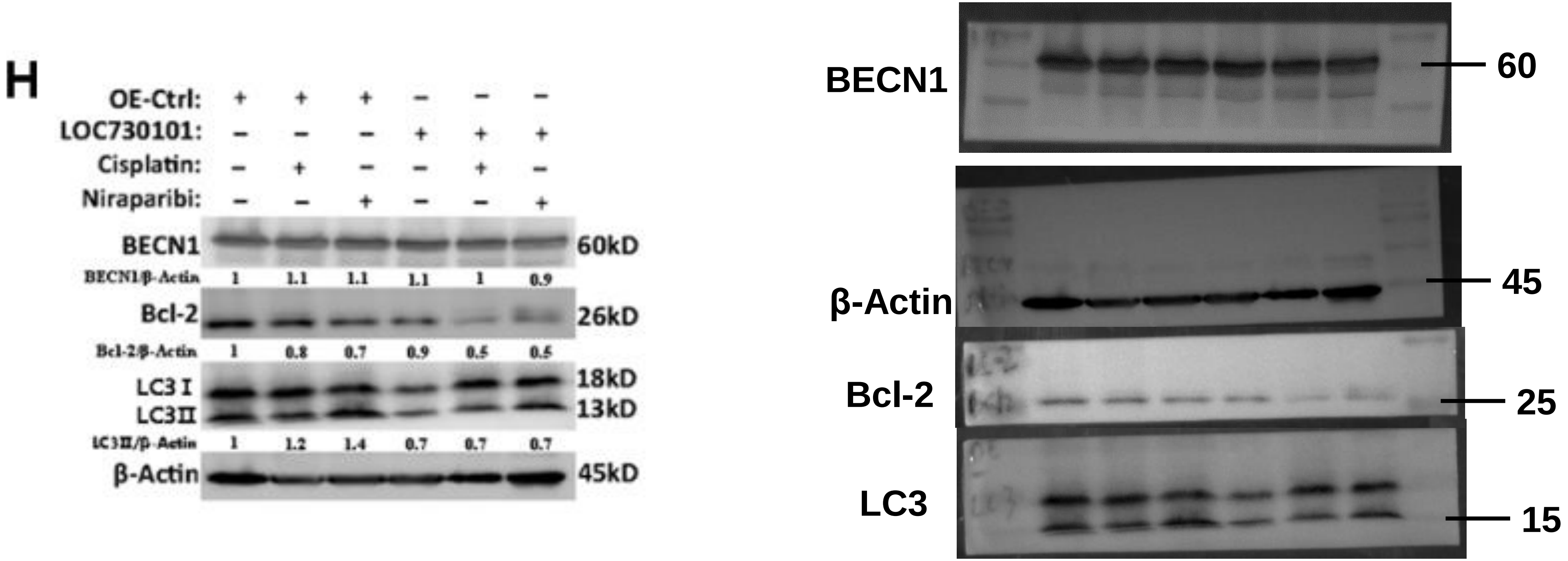
H
OE-Ctrl: + + + − − −
LOC730101: − − − + + +
Cisplatin: − + − − + −
Niraparibi: − − + − − +
BECN1
60kD
BECN1/β-Actin 1 1.1 1.1 1.1 1 0.9
Bcl-2
26kD
Bcl-2/β-Actin 1 0.8 0.7 0.9 0.5 0.5
LC3 I
LC3 II
18kD
13kD
LC3II/β-Actin 1 1.2 1.4 0.7 0.7 0.7
β-Actin
45kD
BECN1
60
β-Actin
45
Bcl-2
25
LC3
15

Fig. 4H

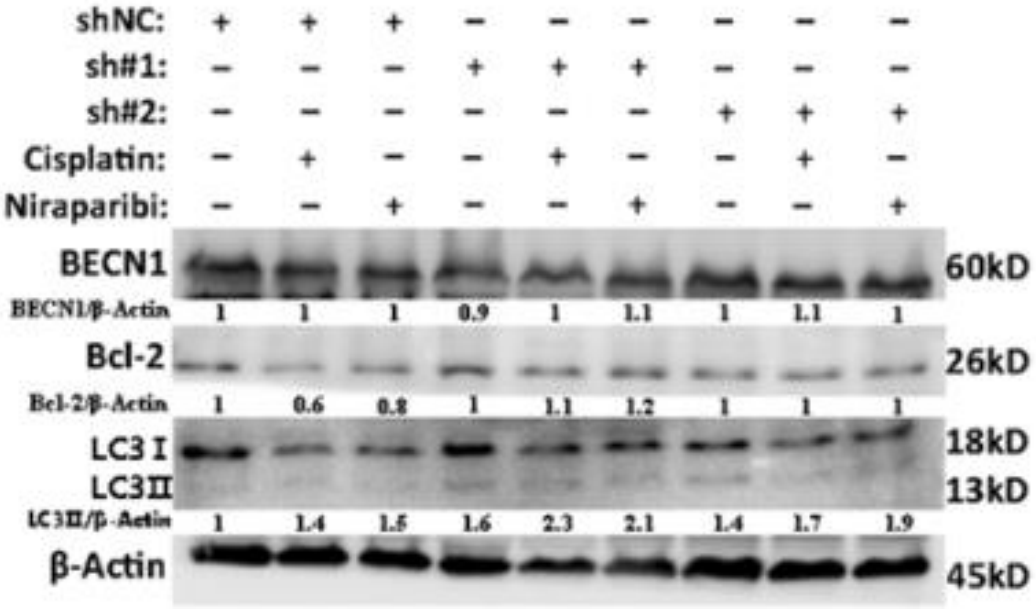

shNC: + + + − − − − − −
sh#1: − − − + + + − − −
sh#2: − − − − − − + + +
Cisplatin: − + − − + − − + −
Niraparibi: − − + − − + − − +
BECN1
60kD
BECN1/β-Actin 1 1 1 0.9 1 1.1 1 1.1 1
Bcl-2
26kD
Bcl-2/β-Actin 1 0.6 0.8 1 1.1 1.2 1 1 1
LC3 I
18kD
LC3II
13kD
LC3II/β-Actin 1 1.4 1.5 1.6 2.3 2.1 1.4 1.7 1.9
β-Actin
45kD

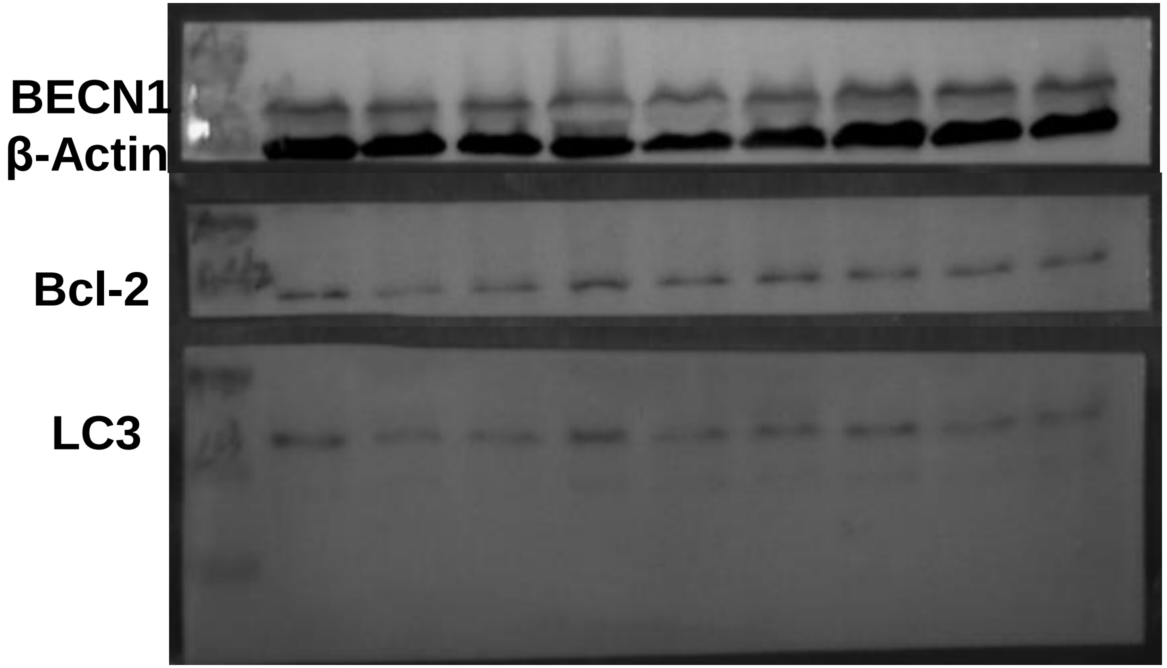

BECN1
β-Actin
Bcl-2
LC3

Fig. 4I

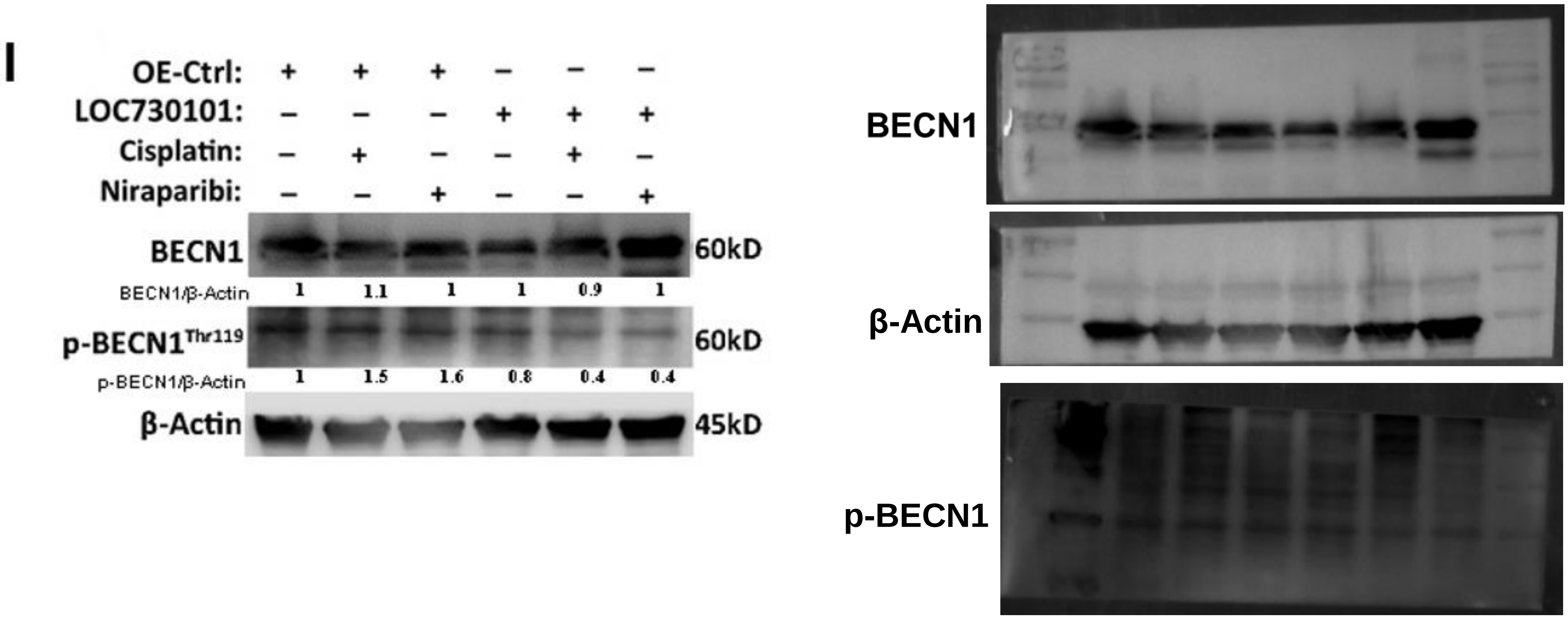
I
OE-Ctrl: + + + − − −
LOC730101: − − − + + +
Cisplatin: − + − − + −
Niraparibi: − − + − − +
BECN1
60kD
BECN1/β-Actin 1 1.1 1 1 0.9 1
p-BECN1Thr119
60kD
p-BECN1/β-Actin 1 1.5 1.6 0.8 0.4 0.4
β-Actin
45kD
BECN1
β-Actin
p-BECN1

Fig. 4I

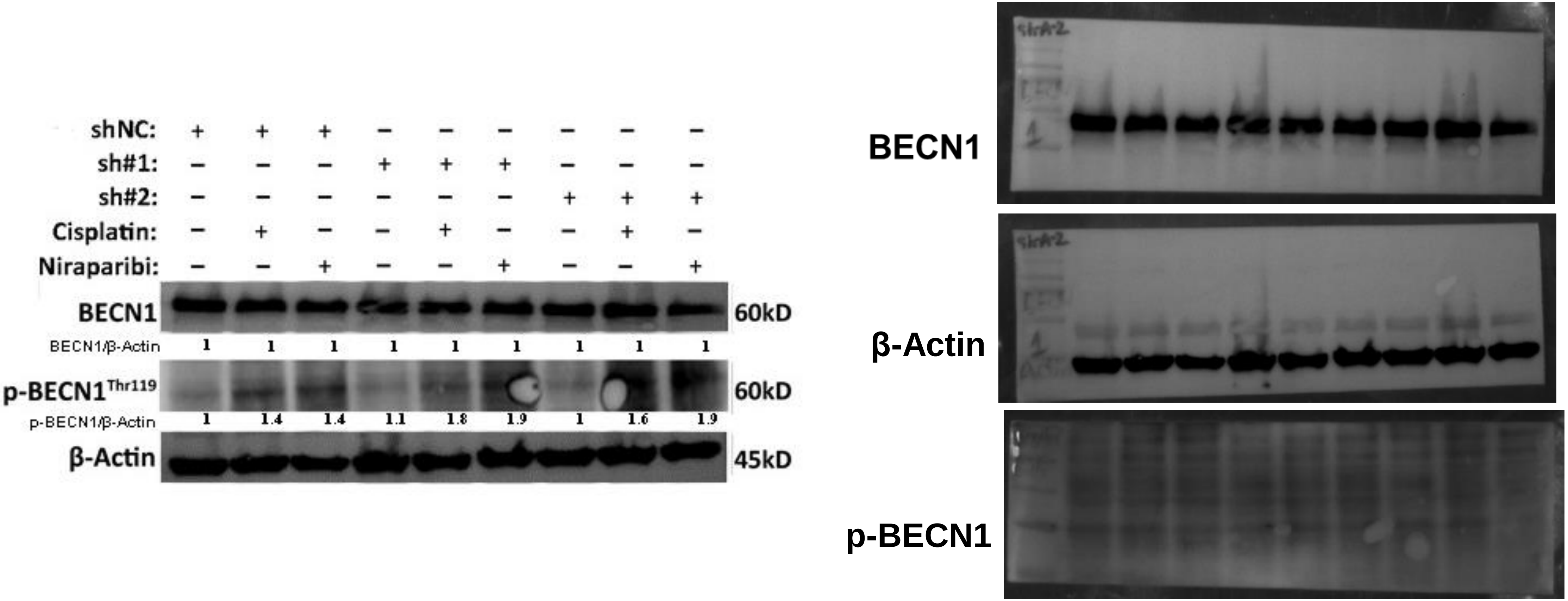

shNC: + + + − − − − − −
sh#1: − − − + + + − − −
sh#2: − − − − − − + + +
Cisplatin: − + − − + − − + −
Niraparibi: − − + − − + − − +
BECN1
60kD
BECN1/β-Actin 1 1 1 1 1 1 1 1 1
p-BECN1Thr119
60kD
p-BECN1/β-Actin 1 1.4 1.4 1.1 1.8 1.9 1 1.6 1.9
β-Actin
45kD
BECN1
β-Actin
p-BECN1

Fig. 4J

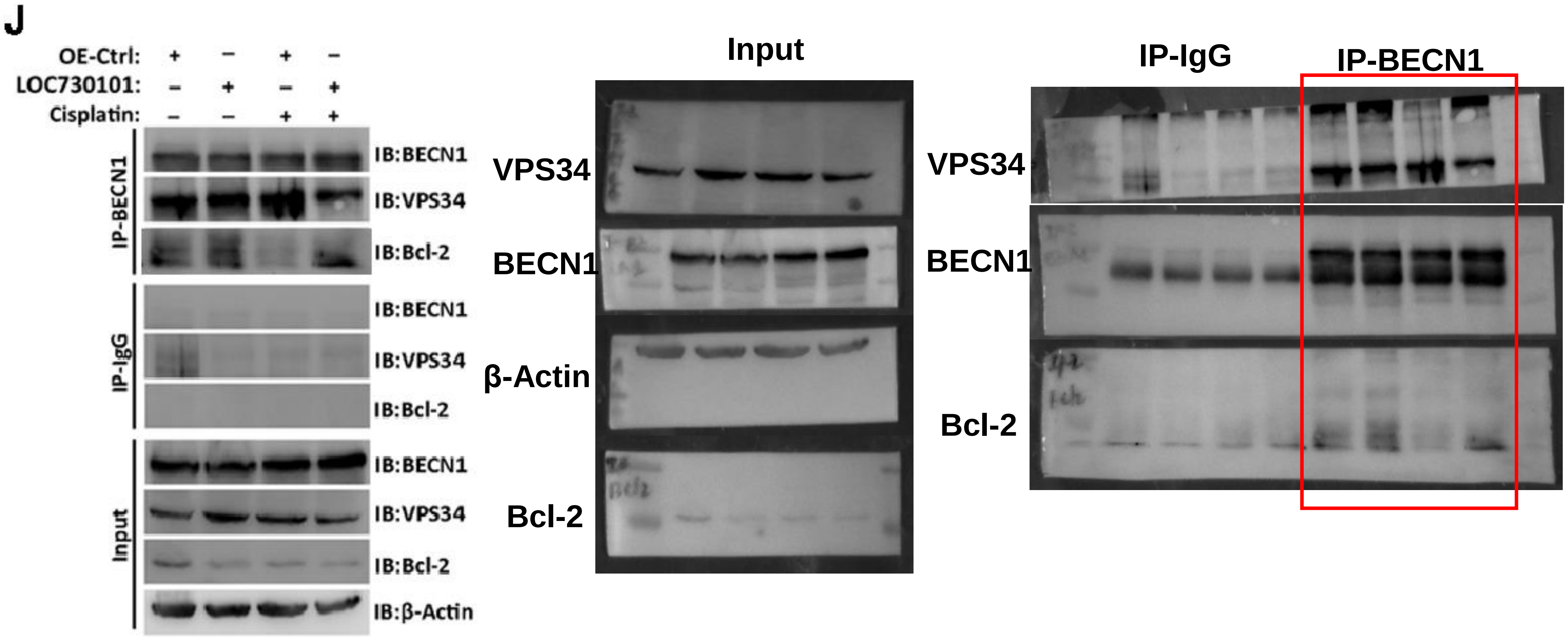
J
OE-Ctrl: + − + −
LOC730101: − + − +
Cisplatin: − − + +
IP-BECN1
IB:BECN1
IB:VPS34
IB:Bcl-2
IP-IgG
IB:BECN1
IB:VPS34
IB:Bcl-2
Input
IB:BECN1
IB:VPS34
IB:Bcl-2
IB:β-Actin
Input
VPS34
BECN1
β-Actin
Bcl-2
IP-IgG
IP-BECN1
VPS34
BECN1
Bcl-2

Fig. 4J

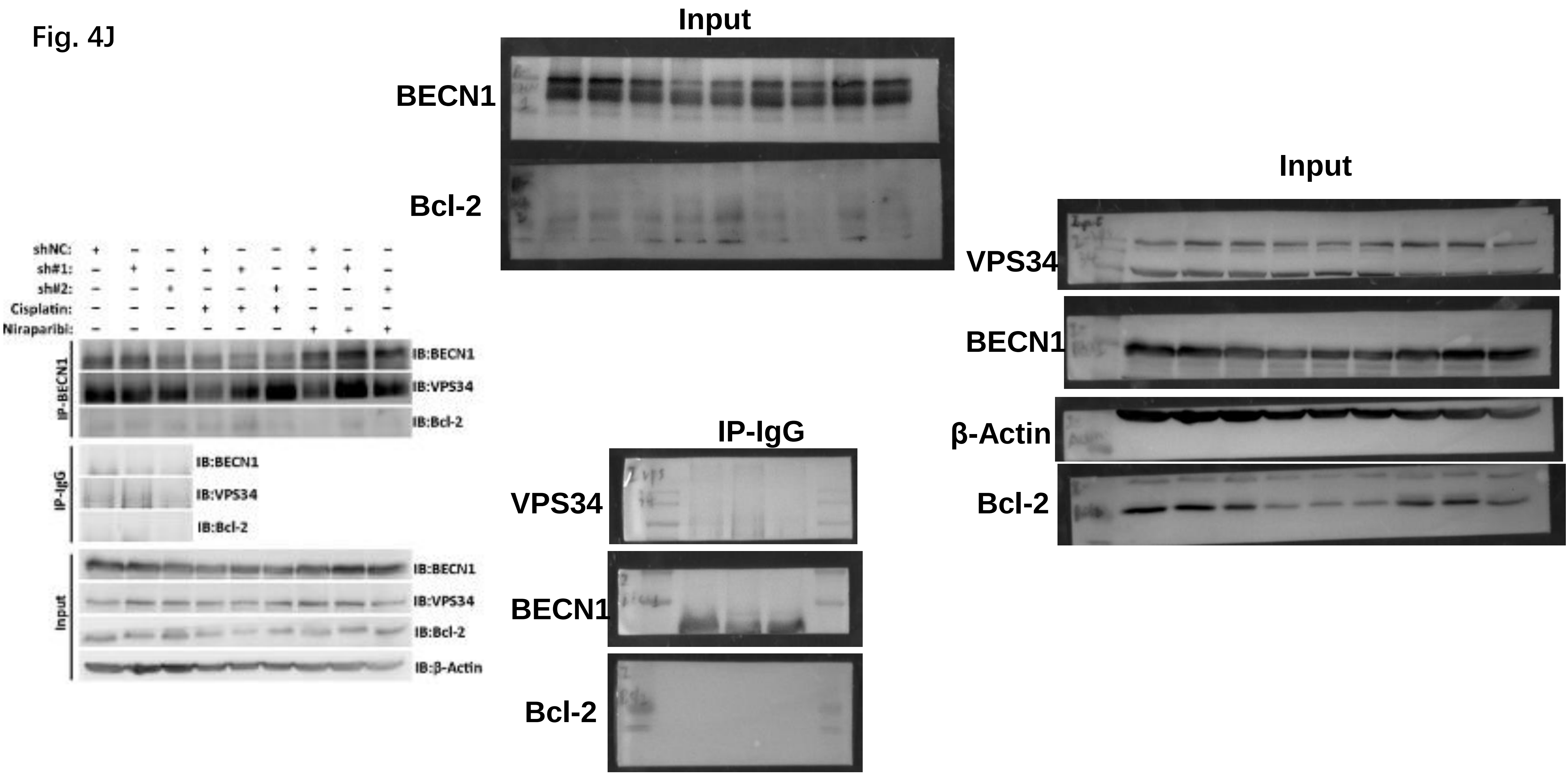
shNC:
sh#1:
sh#2:
Cisplatin:
Niraparibi:
IP-BECN1
IB:BECN1
IB:VPS34
IB:Bcl-2
IP-IgG
IB:BECN1
IB:VPS34
IB:Bcl-2
Input
IB:BECN1
IB:VPS34
IB:Bcl-2
IB:β-Actin
Input
BECN1
Bcl-2
IP-IgG
VPS34
BECN1
Bcl-2
Input
VPS34
BECN1
β-Actin
Bcl-2

Fig. 5B

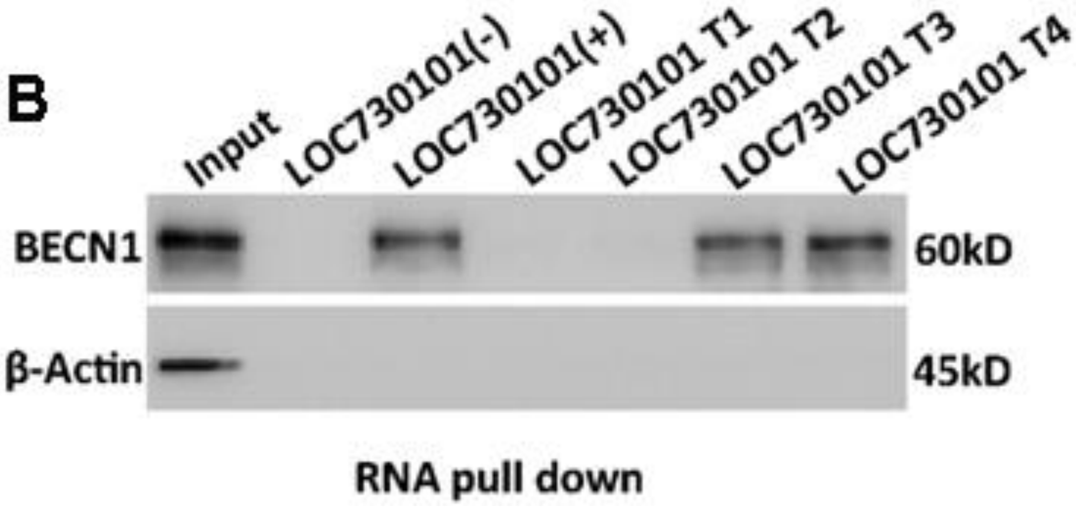
B
Input
LOC730101(-)
LOC730101(+)
LOC730101 T1
LOC730101 T2
LOC730101 T3
LOC730101 T4
BECN1
60kD
β-Actin
45kD
RNA pull down

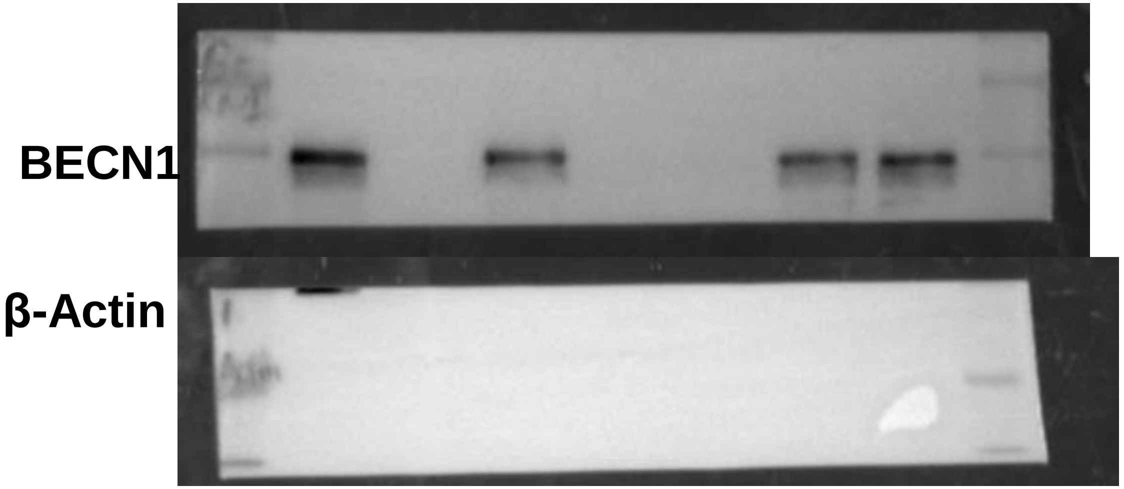
BECN1
β-Actin

Fig. 5F

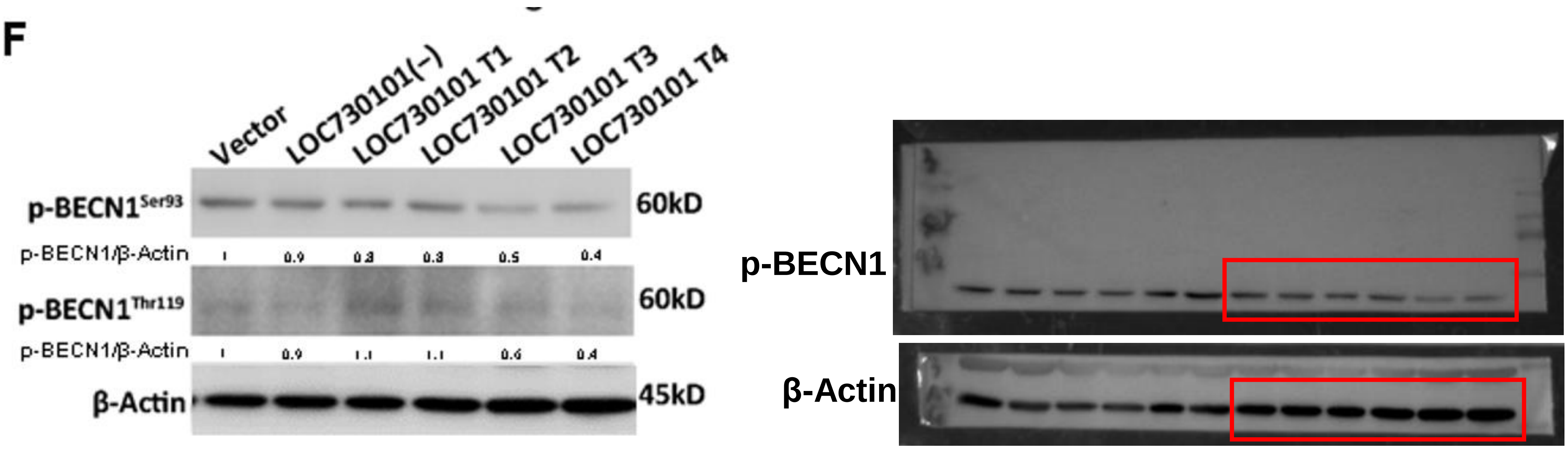
F
Vector
LOC730101(-)
LOC730101 T1
LOC730101 T2
LOC730101 T3
LOC730101 T4
p-BECN1Ser93
60kD
p-BECN1/β-Actin
1
0.9
0.8
0.8
0.5
0.4
p-BECN1Thr119
60kD
p-BECN1/β-Actin
1
0.9
1.1
1.1
0.6
0.4
β-Actin
45kD
p-BECN1
β-Actin

Fig. 6A

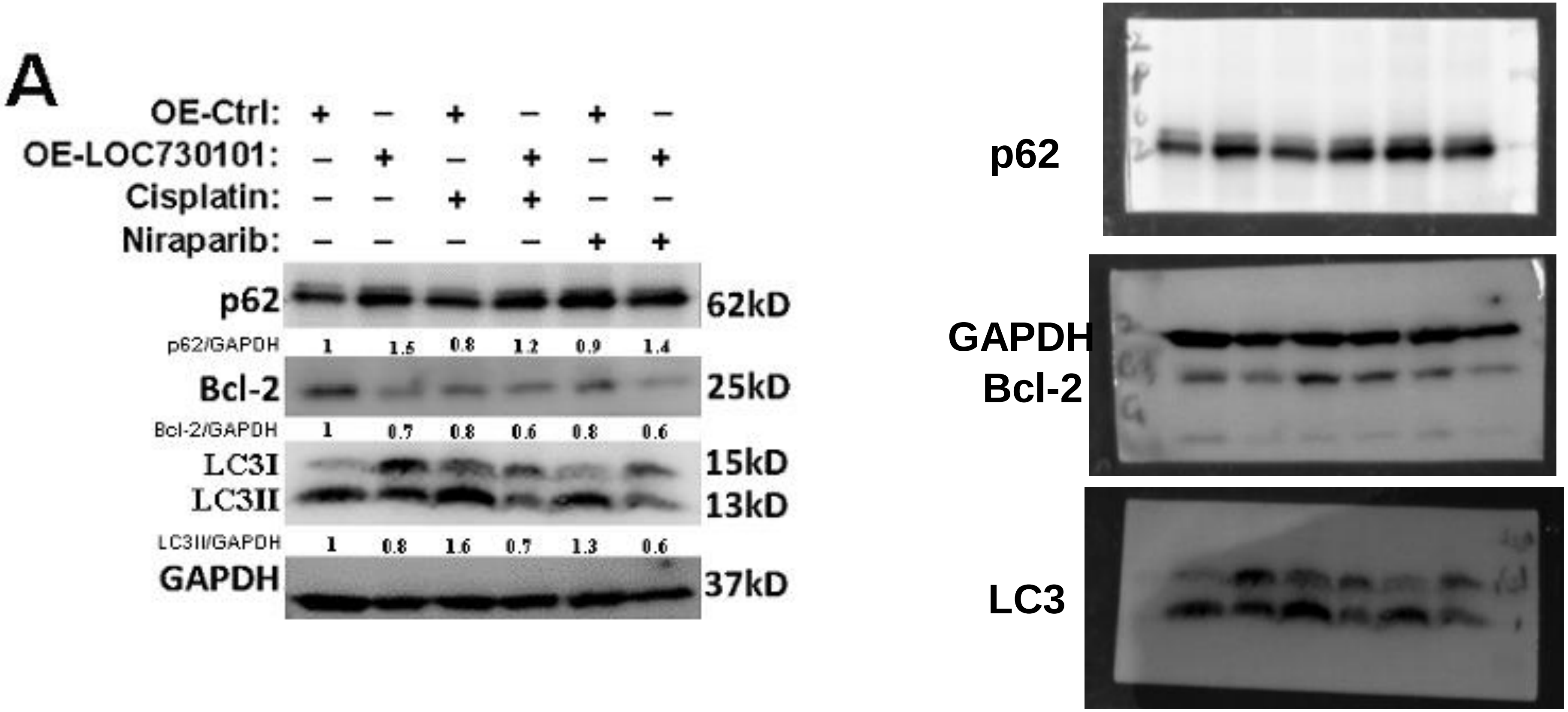
A
OE-Ctrl: + − + − + −
OE-LOC730101: − + − + − +
Cisplatin: − − + + − −
Niraparib: − − − − + +
p62
62kD
p62/GAPDH 1 1.5 0.8 1.2 0.9 1.4
Bcl-2
25kD
Bcl-2/GAPDH 1 0.7 0.8 0.6 0.8 0.6
LC3I
LC3II
15kD
13kD
LC3II/GAPDH 1 0.8 1.6 0.7 1.3 0.6
GAPDH
37kD
p62
GAPDH
Bcl-2
LC3

Fig. 6B

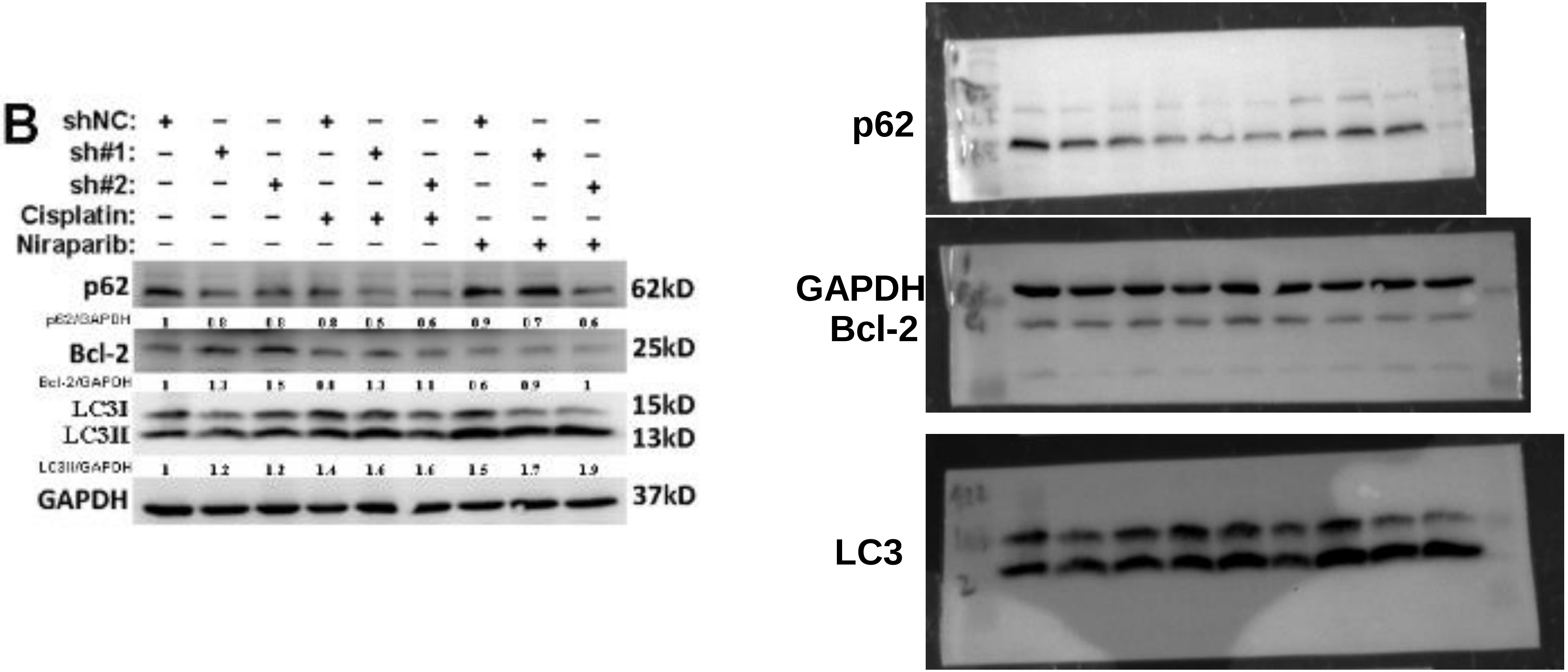
B
shNC:
sh#1:
sh#2:
Cisplatin:
Niraparib:
p62
62kD
p62/GAPDH
Bcl-2
25kD
Bcl-2/GAPDH
LC3I
15kD
LC3II
13kD
LC3II/GAPDH
GAPDH
37kD
p62
GAPDH
Bcl-2
LC3

Fig. 7E

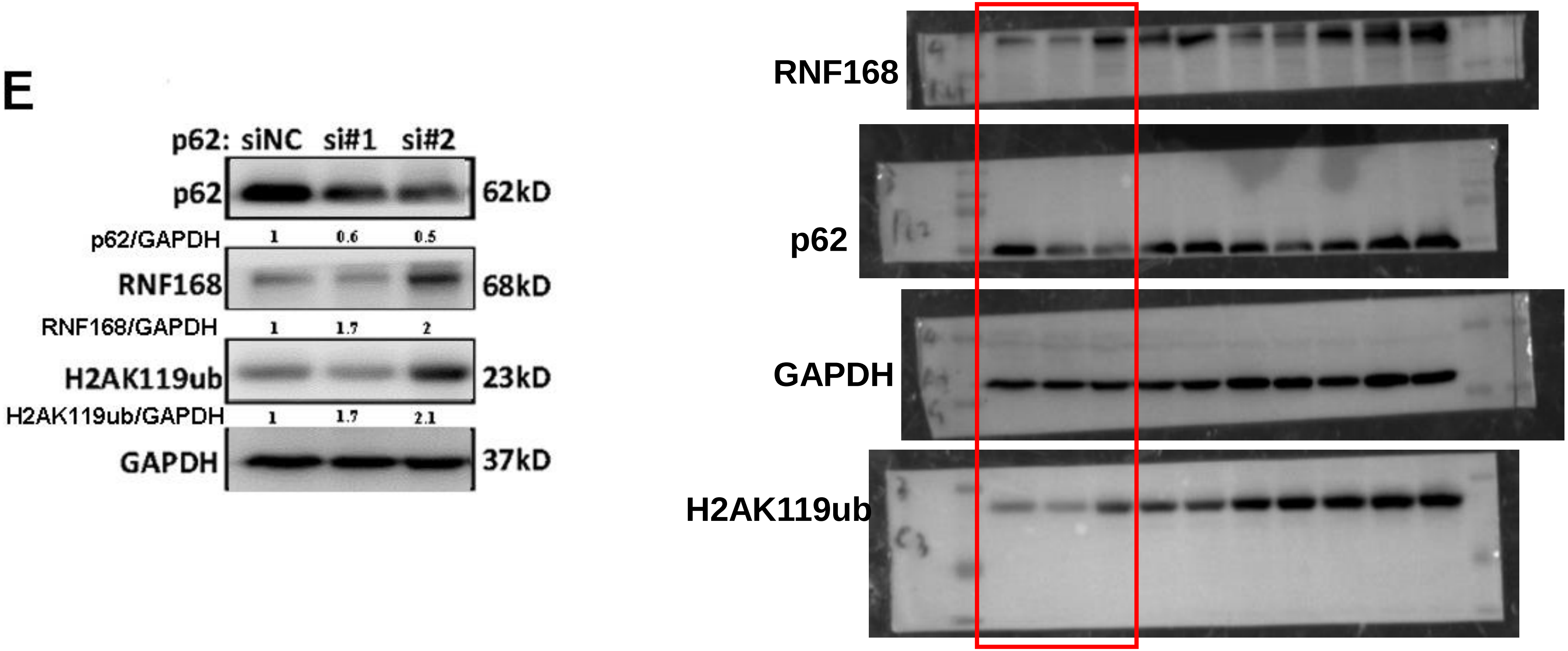
E
p62: siNC si#1 si#2
p62
62kD
p62/GAPDH 1 0.6 0.5
RNF168
68kD
RNF168/GAPDH 1 1.7 2
H2AK119ub
23kD
H2AK119ub/GAPDH 1 1.7 2.1
GAPDH
37kD
RNF168
p62
GAPDH
H2AK119ub

Fig. 7F

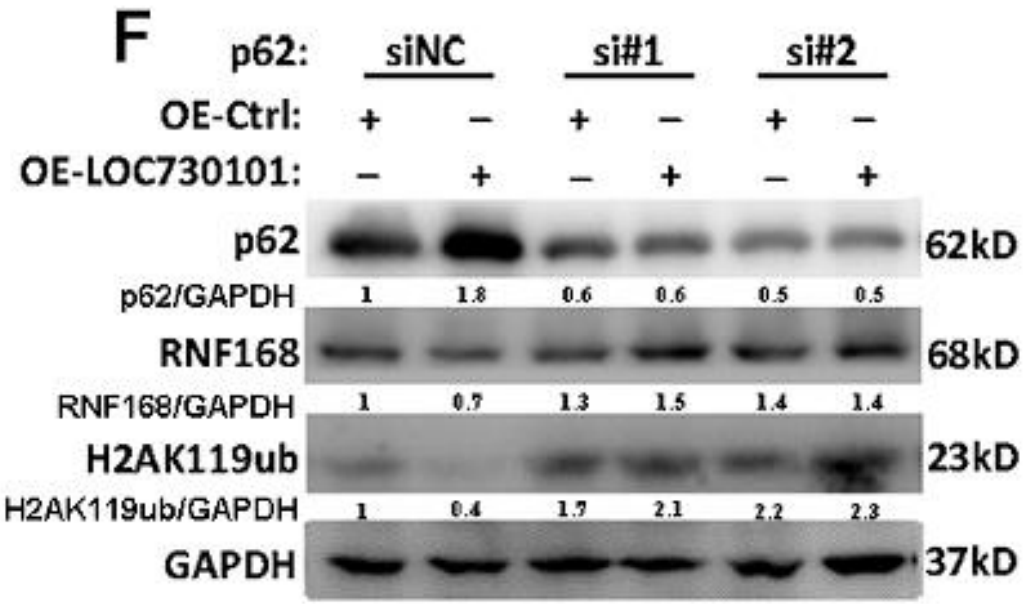
F
p62:
siNC
si#1
si#2
OE-Ctrl:
OE-LOC730101:
p62
p62/GAPDH
1
1.8
0.6
0.6
0.5
0.5
RNF168
RNF168/GAPDH
1
0.7
1.3
1.5
1.4
1.4
H2AK119ub
H2AK119ub/GAPDH
1
0.4
1.7
2.1
2.2
2.3
GAPDH
62kD
68kD
23kD
37kD

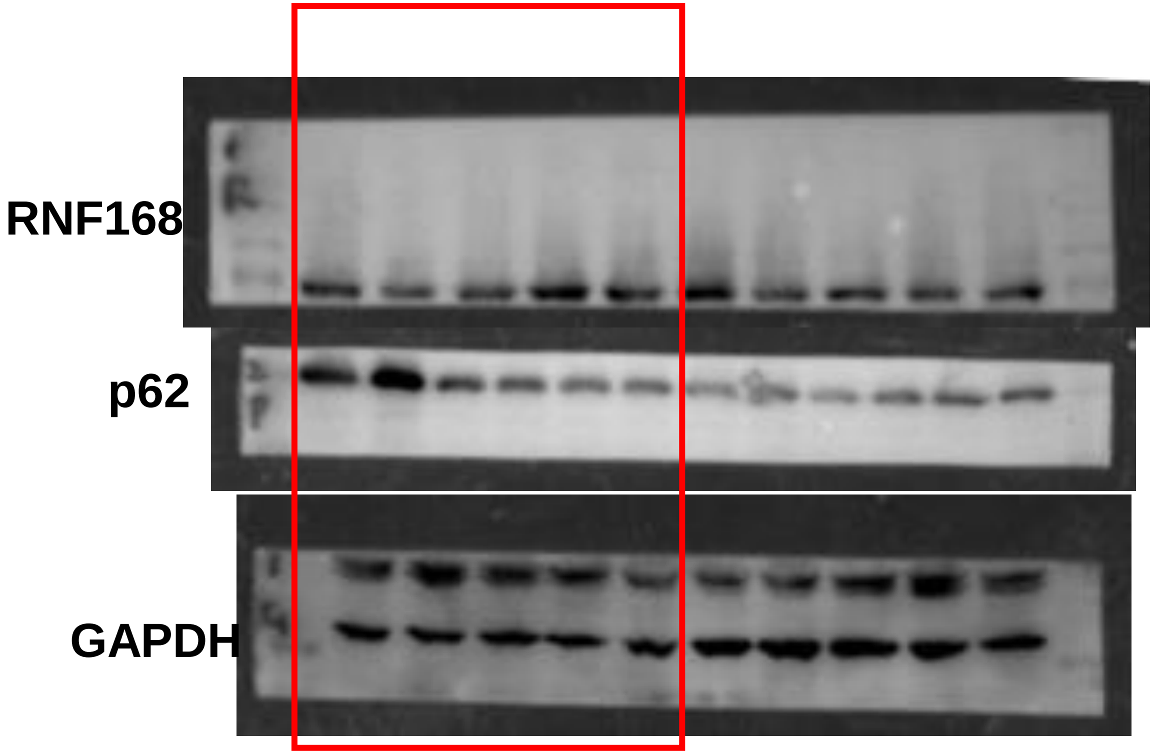
RNF168
p62
GAPDH

Fig. 7G

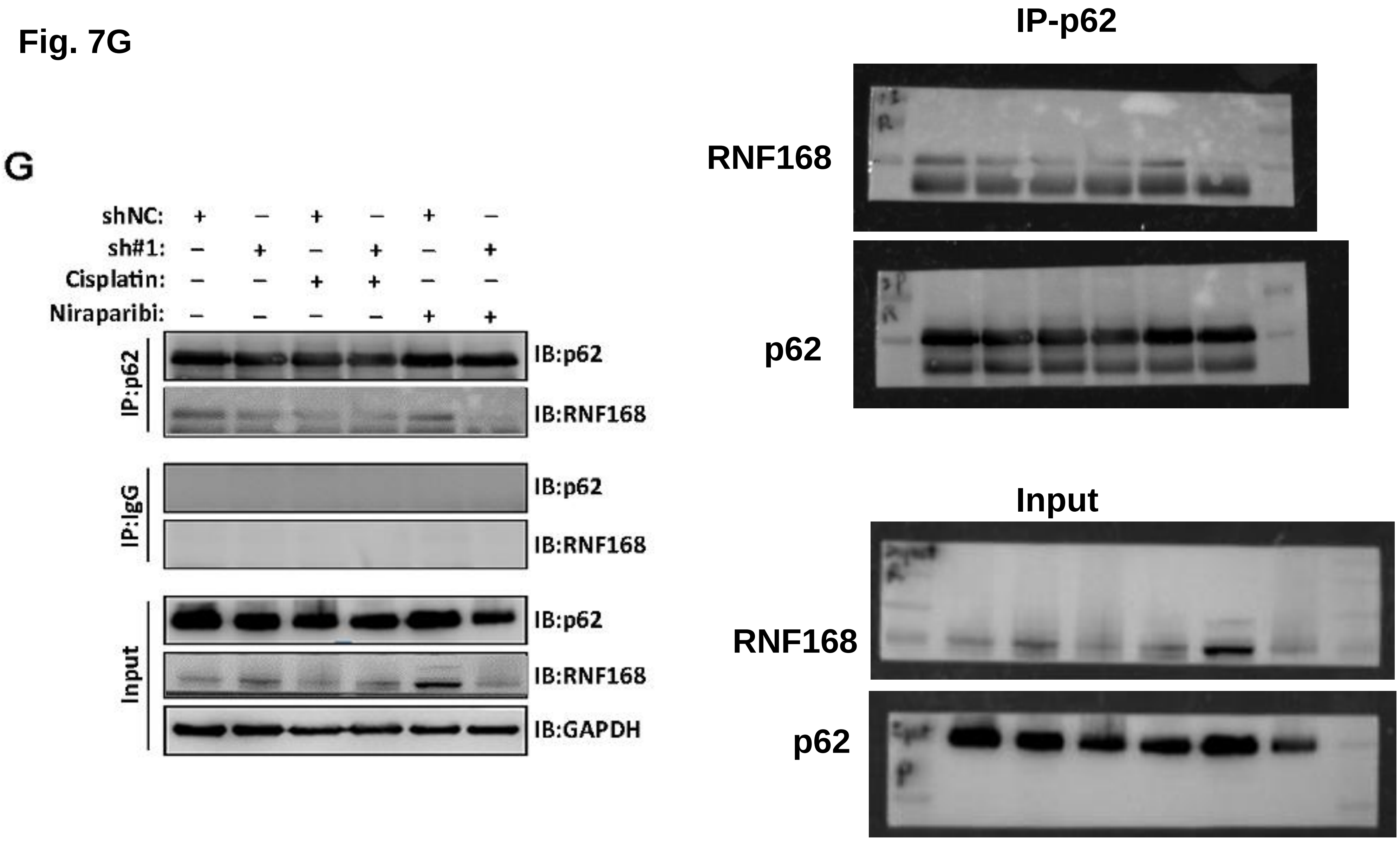
G
shNC: + − + − + −
sh#1: − + − + − +
Cisplatin: − − + + − −
Niraparibi: − − − − + +
IP:p62
IB:p62
IB:RNF168
IP:IgG
IB:p62
IB:RNF168
Input
IB:p62
IB:RNF168
IB:GAPDH
IP-p62
RNF168
p62
Input
RNF168
p62
